# Non-Poissonian Bursts in the Arrival of Phenotypic Variation Can Strongly Affect the Dynamics of Adaptation

**DOI:** 10.1093/molbev/msae085

**Published:** 2024-05-02

**Authors:** Nora S Martin, Steffen Schaper, Chico Q Camargo, Ard A Louis

**Affiliations:** Rudolf Peierls Centre for Theoretical Physics, University of Oxford, Oxford OX1 3PU, UK; Rudolf Peierls Centre for Theoretical Physics, University of Oxford, Oxford OX1 3PU, UK; Rudolf Peierls Centre for Theoretical Physics, University of Oxford, Oxford OX1 3PU, UK; Faculty of Environment, Science and Economy, University of Exeter, Exeter EX4 4QF, UK; Rudolf Peierls Centre for Theoretical Physics, University of Oxford, Oxford OX1 3PU, UK

**Keywords:** evolution, genotype–phenotype map, population genetics

## Abstract

Modeling the rate at which adaptive phenotypes appear in a population is a key to predicting evolutionary processes. Given random mutations, should this rate be modeled by a simple Poisson process, or is a more complex dynamics needed? Here we use analytic calculations and simulations of evolving populations on explicit genotype–phenotype maps to show that the introduction of novel phenotypes can be “bursty” or overdispersed. In other words, a novel phenotype either appears multiple times in quick succession or not at all for many generations. These bursts are fundamentally caused by statistical fluctuations and other structure in the map from genotypes to phenotypes. Their strength depends on population parameters, being highest for “monomorphic” populations with low mutation rates. They can also be enhanced by additional inhomogeneities in the mapping from genotypes to phenotypes. We mainly investigate the effect of bursts using the well-studied genotype–phenotype map for RNA secondary structure, but find similar behavior in a lattice protein model and in Richard Dawkins’s biomorphs model of morphological development. Bursts can profoundly affect adaptive dynamics. Most notably, they imply that fitness differences play a smaller role in determining which phenotype fixes than would be the case for a Poisson process without bursts.

## Introduction

Darwinian evolution accomplishes change over time through the joint processes of variation and selection. There is a longstanding tradition that focuses on the second step of the evolutionary process, using population genetics calculations that describe how genetic drift and natural selection affect the fixation dynamics in a population that initially starts with multiple alleles with different fitness, but where no new alleles appear (Gillespie 2004). It is also possible to include the first step, the introduction of novel phenotypic variation, within a population genetics framework (McCandlish and Stoltzfus 2014). Since the fitness value of an allele is fundamentally caused by the interaction of the phenotype it represents with the environment, one can think of alleles with different fitness as representing different phenotypes. A common underlying assumption in this class of models is that the introduction of new alleles can be characterized by an average rate (see, e.g. Yampolsky and Stoltzfus 2001; McCandlish and Stoltzfus 2014). While these rates can differ, these models assume that individual introductions are uncorrelated, leading to Poisson statistics. We will call models that make such assumptions *average-rate models*.

A more sophisticated way to treat the introduction of novel phenotypic variation is to consider a genotype–phenotype (GP) map that explicitly models how random mutations lead to new phenotypes (Alberch 1991), see, e.g. Ahnert (2017) and Manrubia et al. (2021) for recent reviews. Examples of well-studied GP maps include RNA secondary structures (Schuster et al. 1994; Aguirre et al. 2011; Capitan et al. 2015), simplified models of protein structure, such as the hydrophobic-polar (“HP”) lattice model of tertiary structure (Li et al. 1996) and the tile-based “polyomino” model of protein quaternary structure (Greenbury et al. 2014), and gene regulatory networks (Ciliberti et al. 2007; Camargo and Louis 2020; Catalán et al. 2020). These models describe different biological entities, but all create a bridge between two levels. At the first level of “genotypes”, information is genetically encoded, for example, in nucleic acid sequences or amino acid sequences, and can be directly changed through mutations. The second level of “phenotypes” describes higher-order characteristics of biological or functional relevance whose evolution we are interested in, for example, molecular structures or patterns of gene expression. The well-studied examples listed above all focus on the molecular scale due to the computational complexity of modeling larger-scale phenotypes. However, the framework of GP maps can be applied more broadly (Martin et al. 2024), for example, to Richard Dawkins’s biomorphs (Dawkins 1989, 2016), a simple model of development.

Interestingly, despite the diversity of the biological entities they represent, all these GP maps exhibit certain commonalities (Ahnert 2017; Manrubia et al. 2021). For example, mutations can be neutral, implying that a given phenotype can be generated by multiple distinct genotypes (Schuster et al. 1994). These then form the “neutral set” of that phenotype. Neutral sets of genotypes are not randomly distributed but are thought to display “neutral correlations” (Greenbury et al. 2016). For example, two genotypes that differ by a single mutation are much more likely to correspond to the same phenotype and thus the same neutral set than two randomly chosen genotypes. This implies that a population can drift from genotype to genotype within a neutral set (Wagner 2008; Greenbury et al. 2016). There is a small caveat, namely that the whole neutral set is not always connected through neutral mutations, due in part to biophysical constraints (for example, in RNA, one needs a double mutation to change a CG bond to a GC bond; van Nimwegen et al. 1999; Schaper et al. 2012) so that the neutral set consists of several disjoint parts, which are referred to as *neutral components* (NCs; Schaper et al. 2012).

One important motivation for including the complexity of a GP map is to study the dynamics of neutral evolution on a NC (Bastolla et al. 1999; van Nimwegen et al. 1999; Capitan et al. 2015; Smerlak 2021). For example, different genotypes in a NC can have different robustness (i.e. a different number of neutral mutations per genotype; Aguirre et al. 2011). These inhomogeneities in the robustness imply that the supply of neutral mutations is genotype-dependent and thus changes over evolutionary time, which can lead to overdispersion in the rate of neutral fixations (Takahata 1987; Bastolla et al. 1999; Wilke 2004; Manrubia and Cuesta 2015).

**Fig. 1.**
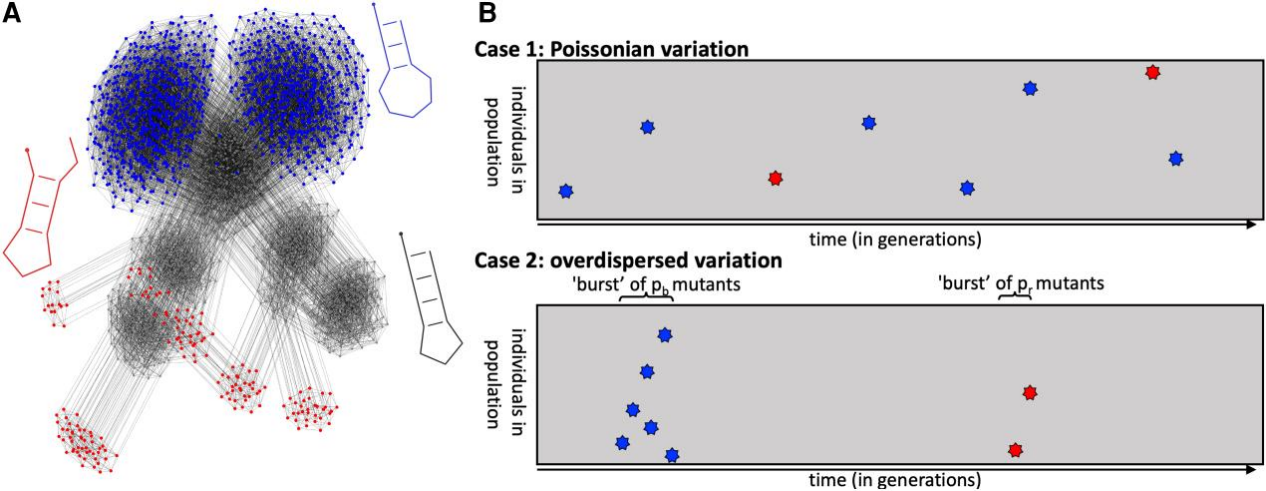
Structure in the mapping from genotypes to phenotypes can induce non-Poissonian bursts in the introduction of novel variation. a) Genotypes of sequence length L=12nt mapping to three selected RNA secondary structure phenotypes (shown in gray, red, and blue) are drawn as a mutational network. Each genotype is a network node and each gray edge between two genotypes means that these two genotypes are only one point mutation apart. A full NC of 1,094 genotypes (nodes) is shown for the gray phenotype (specifically the NC containing the sequence AUACGAAACGUA), while only those nodes connected to the gray NC are shown for the red and blue phenotypes. This network is heterogeneous in several ways (see Schaper and Louis 2014; Greenbury 2014; Capitan et al. 2015; Weiß and Ahnert 2020; Greenbury et al. 2016; McCandlish 2012): first, not all gray genotypes are portal genotypes for red and blue, i.e. genotypes with mutational connections to red or blue phenotypes. Second, the gray NC has a community structure where the nodes form several densely connected clusters. Third, the portal genotypes to the red or blue phenotype are concentrated on a few regions of the gray network, i.e. transitions to blue or red are very likely from some gray genotypes and their mutational neighbors, but impossible otherwise. b) Idealized schematic of individuals in the population (*y*-axis) vs. time (*x*-axis). The population starts on the gray phenotype and moves through the gray NC by neutral mutations. Other novel phenotypes can appear through random mutations, but in this simplified case of strong stabilizing selection, the novel phenotypes only appear for one generation. Here only two novel phenotypes, blue and red, are depicted, with blue appearing at a larger rate than red. Case 1 depicts the classical picture with Poisson statistics, whereas case 2 illustrates “bursty/overdispersed variation” due to the heterogeneous structure in the GP map. Both cases have the same average rates of introduction. Note that each color stands for one *phenotype*, but in this many-to-one mapping, this does not imply that they have the same *genotype*. This and the fact that we focus on burstiness in the newly *introduced* phenotypes, not in the times that phenotypes are *fixed* in the population, are differences from the overdispersion of the molecular clock in neutral evolution.

The effect of the GP map structure on neutral evolutionary dynamics prompts the question of whether inhomogeneities present in the GP map can also shape the introduction of novel phenotypes. To illustrate this point, Fig. 1a shows a NC taken from the RNA secondary structure GP map for a phenotype $p_{g}$ (gray) and the point mutation links it makes to two other phenotypes, $p_{r}$ (red) and $p_{b}$ (blue). Let us focus on the sequences or genotypes with a particular novel phenotype $p_{i}$ in their mutational neighborhood, the *portal genotypes*  $g_{\;p_{i}}$ for $p_{i}$ (similar to Crutchfield 2003). A population can only produce $p_{i}$ as variation if such portal genotypes $g_{\;p_{i}}$ are present in the population (except at very high mutation rates when double mutations occur more frequently). Thus, we expect to observe multiple appearances of a specific phenotype $p_{i}$ when a portal is present in a population (a burst), and no appearances of $p_{i}$ otherwise (see the schematic in Fig. 1b; also Schaper and Louis 2014). This simple argument suggests that non-Poissonian patterns in the appearance of a new phenotype $p_{i}$ will occur whenever only some genotypes on a NC are portals to $p_{i}$.

There are additional types of inhomogeneities in NCs that may further contribute to bursty $p_{i}$ introductions. For example, genotypes in the NC of an initial phenotype $p_{g}$ typically form clusters (or communities from a network science perspective) where genotypes in the same cluster are more highly connected through mutations (Greenbury 2014; Capitan et al. 2015; Weiß and Ahnert 2020). This structure could trap evolving populations in one part of a NC, and thus amplify other inhomogeneities (McCandlish 2011). Finally, we see in Fig. 1a that portal genotypes to a given $p_{i}$ are typically clustered within a NC: the probability that a portal genotype has multiple mutations to $p_{i}$ or that its close neighbors are also portals is higher than in a random model (McCandlish 2012; Greenbury et al. 2016). This clustering of mutations to $p_{i}$ around specific parts of a NC is referred to as *non-neutral correlations* (Greenbury et al. 2016). Taken together, the number of mutational connections to $p_{i}$ is typically highly inhomogeneous over a NC, and this inhomogeneity is expected to lead to “bursty” appearances of $p_{i}$ in populations evolving on that NC (McCandlish 2012; Schaper and Louis 2014), for which some evidence exists for specific NCs (McCandlish 2012).

Overdispersion in molecular evolution has already been discussed in other contexts, for example, Ohta and Kimura’s (1971) and Gillespie’s (1989) famous work on the overdispersion of the molecular clock in the 1970s and 1980s. However, the bursts we describe here refer to a distinct, although not mutually exclusive phenomenon. Here, we show how the *appearance* of a *particular novel phenotype* can exhibit overdispersion (see Fig. 1b). These appearances can originate from different substitutions and thus different *genotypes*. For example, in Fig. 1a, from a given gray node, several mutational connections can lead to distinct nodes corresponding to the “blue” phenotype. These bursts can affect the timing and probability of different fixation processes, but they cannot lead to a burst in *fixations* since each novel phenotype can only go into fixation once. Thus, there are two key differences between the “bursts” described in this paper and observations of an overdispersed molecular clock: the former can only be observed on the *phenotypic* level and in the *introduction* of new variation, whereas the latter can be observed from *sequence* data and from the timing of *fixed* mutations. More recent observations of overdispersed phenomena in neutral and adaptive molecular evolution typically also fall into this latter category: overdispersion is observed in substitutions on the *sequence* level that reach a certain frequency threshold in the population (i.e. not counting introductions that are immediately lost through drift). Examples include “bursts” of substitutions in the influenza hemagglutinin protein, which may be due to fluctuations in coalescent tree structures without recombination (Kim and Kim 2015), or due to hitchhiking effects (Strelkowa and Lässig 2012), which have also been invoked in overdispersed mutations at lower frequencies in evolving yeast populations (Lang et al. 2013).

A second association with the term “burst” is with the concept of “punctuated equilibrium” (Gould 2002). The overdispersed variation analyzed here could indeed lead to punctuated patterns, but even an average-rate model is sufficient for modeling long periods of stasis until a rare phenotypic transition $p_{i}$ appears and goes into fixation (see, for example, Bakhtin et al. 2021). In previous work showing punctuated dynamics on GP maps (Huynen et al. 1996; Fontana and Schuster 1998a; Koelle et al. 2006), both of these factors are likely to have played a role. These examples include one influential model of influenza evolution (Koelle et al. 2006), which sought to explain a phenotypically bursty, but genotypically continuous pattern of evolution, i.e. a similar phenomenon as in our analysis here. Despite this connection, our bursts are predicted to be more prominent in a mutation-limited regime, and may thus not be relevant to rapidly mutating influenza viruses.

In this paper, we will explore the conditions for phenotypic “bursts” to occur and their effects on evolutionary dynamics, using the RNA sequence-to-secondary-structure GP map as a main example. This is a famous and much-studied GP map model (Manrubia et al. 2021) because it is both biologically relevant for non-coding RNAs and can be efficiently modeled with computational techniques using, for example, the ViennaRNA package (Lorenz et al. 2011). To check our results beyond this particular GP map model, we also examine bursts for two further GP maps: the HP lattice model for protein tertiary structure (Dill 1985; Li et al. 1996) and the biomorphs model of development (Dawkins 1989, 2016).

We proceed as follows: first, we build on simple scaling arguments from Schaper and Louis (2014) to explore the time scales and sizes of bursts, as well as their impact on adaptive dynamics for the simplest case of the fully monomorphic regime. Next, using a mixture of analytic and computational methods, we separate out the effects of different types of GP map inhomogeneities by constructing a hierarchy of null models. The simplest two models are the average-rate model and a random null model from Schaper and Louis (2014) that has sequences randomly linked to phenotypes. The more complex models add increasing levels of non-random structure until the final level describes the full GP map. All levels of complexity are studied by population genetic simulations, and for the two simplest levels, we can also derive analytic descriptions of the statistics at which novel phenotypes appear through mutations. We repeat the simulations for a range of population sizes and mutation rates and find that, as expected (Schaper and Louis 2014), the introduction of new phenotypes is most strongly overdispersed for large population sizes and low mutation rates. Next, we study how bursts affect adaptive evolution in a landscape where one of the non-neutral variants has a selective advantage over the initial phenotype. We show that bursty dynamics can strongly increase average fixation times compared to an average-rate model. Moreover, the fixation rates saturate at a modest fitness threshold and only weakly increase with fitness above this threshold. The root cause of these effects is that, with bursts, the discovery of a portal genotype is the rate-limiting step in the adaptive dynamics. Finally, we study the *arrival of the frequent* (Schaper and Louis 2014; or *“first come, first served”*; Yampolsky and Stoltzfus 2001) scenario for a two-peaked fitness landscape, where the fitter phenotype has a much lower average rate of appearance. We show that the probability that the fitter, but less frequent, phenotype, fixes first can be markedly suppressed compared to the predictions of average-rate models such as those used in Yampolsky and Stoltzfus (2001), Gomez et al. (2020), and de Aquino Soares et al. (2021), and argue that these effects can extend to more complex fitness landscapes.

## Scaling Arguments for Bursts in the Monomorphic Regime

In this section, we explore some simple scaling arguments to provide intuition for how inhomogeneities in the distribution of portal genotypes, sequences with at least one mutation to a desired phenotype, affect evolutionary dynamics.

We first ask whether the fraction of possible genotypes that are portals for any given phenotype is small or large. Consider a system with genotypes that are sequences of length *L* with alphabet size *K* (see definitions in Table 1). The number of mutational neighbors of any sequence is L(K−1), which grows linearly with *L*. The total number of phenotypes often exceeds this value, and then only a fraction of sequences can be portal genotypes to any given phenotype *p*. For long sequences, this can be deduced from scaling arguments since the total number of phenotypes typically scales exponentially with sequence length *L*,^a^ and this quickly becomes much larger than L(K−1), the number of mutational neighbors. Then the fraction of genotypes that are portals to a given phenotype *p* is typically small.

**Table 1:** An overview of key quantities and their definitions

Symbol	Name	Calculation	Reference
*L*	Sequence length	Free parameter	
*u*	Mutation rate (per site and generation)	Free parameter	
*N*	Population size	Free parameter^a^	
$s_{i}$	Selective advantage of phenotype $p_{i}$	Free parameter	
*K*	Alphabet size	Set by the GP map (K=4 for RNA)	
NC	Neutral component	Set by the GP map (defined as the set of genotypes that map to a single phenotype and that are mutually connected by phenotype-preserving mutations)	Schaper et al. (2012)
$\phi_{p_{i}p_{0}}$	Mutation probability from phenotype $p_{0}$ to $p_{i}$	Set by the GP map (calculated as the probability that a mutation on a genotype from the relevant NC of $p_{0}$ produces a phenotype $p_{i}$)	Schaper and Louis (2014)
Portal genotype to $p_{i}$	A genotype that has $p_{i}$ in its mutational neighborhood	Set by the GP map	
$P_{g_{p_{i}}}$	Probability that an arbitrary genotype is a portal to $p_{i}$	Set by the GP map	
*ρ*	Mutational robustness of a NC	Set by the GP map (calculated as the fraction of phenotype-preserving mutations out of all possible mutations on the NC)	Schaper et al. (2012)
$r_{i}$	Rate of $p_{i}$ introductions in the average-rate model	$r_{i}=LNu\times \phi_{p_{i}p_{0}}$	Schaper and Louis (2014)
$t_{i}^{\mathrm{fix}}$	Expected time for $p_{i}$ introduction and fixation	Derived for the average-rate case in supplementary Equation (S9), Supplementary Material online, and for the random GP map in supplementary Equation (S15), Supplementary Material online	
$t_{\mathrm{ne}}$	Mean time between neutral fixations through drift	$t_{\mathrm{ne}}=(uL\rho {)}^{-1}$	Schaper and Louis (2014)
$t_{\mathrm{gene}}$	Time-scale on which every single substitution in a mutational neighborhood occurs once	$t_{\mathrm{gene}}=\frac{K-1}{Nu}$	Schaper and Louis (2014)
*M*	Burst size in the simplest approx. of the random map	$M=\frac{t_{\mathrm{ne}}}{t_{\mathrm{gene}}}=\frac{N}{(K-1)L\rho }$	Schaper and Louis (2014)
$P_{\;p_{1}}^{\mathrm{fix}}$	Single-mutant fixation probability	$P_{\;p_{1}}^{\mathrm{fix}}=\frac{1-\exp\;(-2s_{1})}{1-\exp\;(-2Ns_{1})}$	Etheridge (2011)
$P_{\mathrm{portal}\;p_{1}}^{\mathrm{fix}}$	Probability of $p_{1}$ fixation before the “portal” genotype disappears through a neutral fixation	Derived in supplementary Equation (S14), Supplementary Material online	

^a^ note that in our simple calculations, we don’t distinguish between census size *N* and the effective population size $N_{e}$, but these can be quite different (Gillespie 2004)

In this paper, we focus on the mutational introduction of a novel phenotype $p_{i}$ in a population that is initialized on a NC of a phenotype $p_{g}$ and evolves neutrally from genotype to genotype in this NC due to genetic drift. We start by building on earlier work by McCandlish (2012) and our group Schaper and Louis (2014), where some of the arguments below were, to our knowledge, first mentioned. Let us consider the *weak-mutation* or *monomorphic regime*, where the product of the point mutation rate *u*, sequence length *L*, and population size *N* is small (NuL<1), i.e. only a small number of new mutations occur in the population in any given generation (McCandlish and Stoltzfus 2014). Thus, the population will be localized on a single genotype $g_{0}$ with a time scale of $t_{\mathrm{ne}}$ given by (Schaper and Louis 2014):


(1) $$t_{\mathrm{ne}}\approx \frac{1}{Lu\rho },$$

where the robustness *ρ* is the fraction of mutations on the NC that are neutral.

While a population is localized on a particular genotype $g_{0}$, it experiences mutations at rate *NuL*, which are distributed among the (K−1)L distinct (neutral or non-neutral) mutational neighbors of that genotype. Thus, each *specific* mutational neighbor is produced every $t_{\mathrm{gene}}$ generations with (Schaper and Louis 2014):


(2) $$t_{\mathrm{gene}}=\frac{(K-1)L}{NuL}=\frac{K-1}{Nu}.$$

By taking the ratio of $t_{\mathrm{ne}}$ and $t_{\mathrm{gene}}$, we can estimate how often any 1-mutational neighbor of $g_{0}$ will be produced while the population remains on a genotype $g_{0}$ (Schaper and Louis 2014):


(3) $$M=\frac{t_{\mathrm{ne}}}{t_{\mathrm{gene}}}=\frac{N}{(K-1)\rho L}\approx \frac{N}{L}.$$

The final approximation follows because (K−1) is 3 (for RNA) and 19 (for proteins) while *ρ* is typically not too small (Greenbury et al. 2016) so that their product is roughly of order 1. We will call *M* the burst size since it is the expected number of times the same new genotype (and thus the same new phenotype $p_{i}$) is introduced while the population is on a portal genotype. The true burst size will be larger if there is more than one mutation to $p_{i}$ in the 1-mutational neighborhood of $g_{0}$, for example, due to the inhomogeneities present in the RNA map. The time scale between such bursts is set by the time-scale for the population to drift onto a new portal genotype, which is long if only a small fraction of genotypes are portals to $p_{i}$. If the probability that a given genotype is a portal genotype to $p_{i}$ is denoted as $P_{g_{\;p_{i}}}$, then the time-scale $t_{\mathrm{port}}$ is


(4) $$t_{\mathrm{port}}\approx \frac{t_{\mathrm{ne}}}{P_{g_{\;p_{i}}}}\gg t_{\mathrm{ne}}.$$

To summarize, in the monomorphic regime, if $P_{g_{\;p_{i}}}\ll 1$ and N/L≫1, there will be long periods with no mutations to $p_{i}$ until the population drifts onto a portal genotype, with a time-scale $t_{\mathrm{port}}$. If this portal genotype has $n_{\;p_{i}}$ mutations to $p_{i}$ in its 1-mutational neighborhood, then (if $p_{i}$ does not fix) the population will produce $p_{i}$ an average of $n_{\;p_{i}}M$ times before the population neutrally fixes to a new genotype on a time-scale $t_{\mathrm{ne}}\ll t_{\mathrm{port}}$. Such a “burst” is illustrated in the second panel of Fig. 1b. Since the appearance of the new phenotype $p_{i}$ depends on a rare event (the fixation of a portal genotype), these appearances will be overdispersed, similarly to the case of noise in gene expression, where a small number of mRNA in a cell may produce a larger number of proteins in bursts (Thattai and Van Oudenaarden 2001).

Perhaps the most interesting impact of bursts is on the dynamics of adaptation. Consider a phenotype $p_{i}$ with a single-mutant fixation probability $P_{\;p_{i}}^{\mathrm{fix}}$. Now, if a portal genotype is found, on average a burst of *M* mutants of phenotype $p_{i}$ will be produced. Then the probability $P_{\mathrm{portal}\;p_{i}}^{\mathrm{fix}}$ that a fixation event occurs by the end of that burst is approximated by the following expression (derivation in supplementary Section S1.3.3, Supplementary Material online):


(5) $$P_{\mathrm{portal}\;p_{i}}^{\mathrm{fix}}={\left(1+\frac{1}{P_{\;p_{i}}^{\mathrm{fix}}M}\right)}^{-1}.$$

**Fig. 2.**
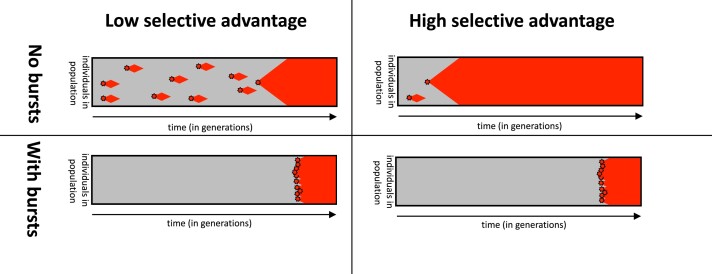
Idealized schematic—the effect of bursts on the time to fixation: in the non-bursty case (first row), the red phenotype $p_{r}$, which is fitter than the initial gray phenotype, appears at intervals that are described by a Poisson process. The fixation time depends strongly on how many appearances of $p_{r}$ are required for its fixation, which in turn depends strongly on its selection coefficient $s_{\;p_{r}}$. In the overdispersed case (second row), there are time intervals where $p_{r}$ does not appear at all for many generations and intervals when the population resides at a portal genotype, and $p_{r}$ is produced many times in quick succession. When $p_{r}$ does not appear at all, it cannot fix, so its selective advantage does not matter. When it appears repeatedly, it is likely to fix as long as its fitness is above a modest threshold given by Equation (6), but how far above the threshold does not matter much. The time to fixation in this regime is thus dominated by the time $t_{\mathrm{port}}$ for the population to reach a portal genotype. The fitness plays a much less important role.

When $P_{\;p_{i}}^{\mathrm{fix}}M\gg 1$, this function saturates toward 1 (see supplementary Fig. S1, Supplementary Material online), and its value is insensitive to changes in the single-mutant fixation probability $P_{\;p_{i}}^{\mathrm{fix}}$, which depends on the selective advantage. In other words, as long as $P_{\;p_{i}}^{\mathrm{fix}}M\gg 1$, a typical burst produces more $p_{i}$ than are strictly needed for fixation. Then, the time to fixation is primarily set by the timing of the first burst rather than by the strength of selection or the size of a burst (Fig. 2). In the case of large bursts with long inter-burst intervals, this can greatly increase the time to fixation compared to an average-rate model with the same mean rate of $p_{i}$ introductions. Since $P_{\;p_{i}}^{\mathrm{fix}}\propto s_{\;p_{i}}$ as long as $1/N\ll s_{\;p_{i}}\ll 1$, where $s_{\;p_{i}}$ is the selection coefficient for $p_{i}$, the saturation effect becomes relevant when


(6) $$s_{\;p_{i}}≳1/M.$$

In the simplest case, where portals have only one instance of $p_{i}$ in their mutational neighborhood, the threshold scales as $s_{p}>L/N$ (see Equations (3) and (6)). For a typical sequence of L≤1,000 bp and a small population of $N∼10^{5}$, this gives $s_{p}∼0.01$. This threshold is remarkably low! Moreover, the threshold can be even smaller if portals have several $p_{i}$ connections due to non-neutral correlations. For simplicity, we have so far worked with an idealized monomorphic population that is always located at one genotype at a time. A fuller treatment of a more realistic monomorphic population is presented in the supplementary Section S1.3, Supplementary Material online, and the resulting predictions are shown alongside our simulation data as cyan lines. But the basic phenomenology is captured by the simple arguments above.

By contrast, in the polymorphic regime NLu≫1, the population will carry a diverse set of genotypes at any time, and so inhomogeneities in the distribution of portal genotypes can be washed out (Schaper and Louis 2014; Greenbury et al. 2016), resulting in dynamics closer to an average-rate model. Nevertheless, as can be seen in Fig. 1a, the inhomogeneities in portal genotypes across a NC can cover a significant range in Hamming distance. Therefore, the strong inhomogeneities may cause bursty behavior further into polymorphic regime than what would be the case for NCs where the only source of inhomogeneity is statistical fluctuations due to a small fraction of portals. Even at extremely high mutation rates, the inhomogeneity of the GP map can be important since high mutation rates typically entail a preference for high-robustness regions of a neutral set, which might be enriched in portals for some phenotypes over others (McCandlish 2012).

Similarly, in what we will call the fast-drift limit N/L<1, where the population is smaller than the genome size, even monomorphic populations will produce new phenotypes with statistics more in line with an average-rate model (Schaper and Louis 2014). The reason is that the population does not produce all genotypes in its one-mutational neighborhood before moving on to a new mutational neighborhood through a neutral fixation (see Equations (1) and (2)). Even for a relatively small population of $N≳10^{5}$ individuals, the fast-drift limit only becomes relevant if we consider sections of the genome longer than $≳10^{5}$ bp, i.e. beyond typical single genes. Again, structural inhomogeneities on larger Hamming distance scales may still cause bursts in this fast-drift regime.

In the next section, we will use a combination of analytic and computational approaches to study in detail how the scaling arguments above apply to a specific system, namely the RNA GP map for sequences of length L=12 nucleotides.

## Results for an RNA GP Map

### A Hierarchy of Simplified Models

**Fig. 3.**
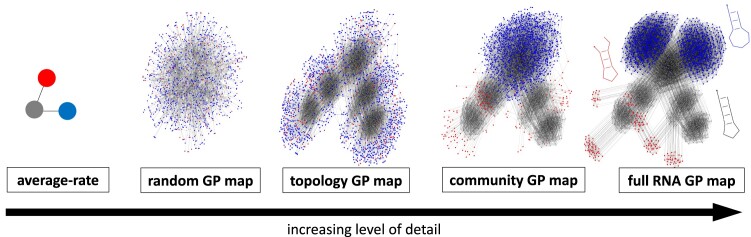
Hierarchy of models with increasing complexity for the RNA GP map. The rightmost network is the same as Fig. 1: the 1,094 genotypes in the initial NC, which corresponds to phenotype $p_{g}$, are drawn as gray nodes and possible point mutation connections are shown as gray lines. In addition to neutral mutations within the NC, mutations to two different non-neutral phenotypes are shown, 1,358 genotypes with phenotype $p_{b}$ (blue nodes) and 176 genotypes with phenotype $p_{r}$ (red nodes). The leftmost model depicts a simple *average-rate model* without the internal structure of a GP map, but the same mean probabilities of mutating to $p_{b}$ and $p_{r}$ (see Schaper and Louis 2014). In the *random GP map*, the probability that a mutation from gray will lead to $p_{i}$ is the same as in the RNA GP map, but otherwise, the assignment between genotypes and phenotypes is random (similar to Schaper and Louis 2014). The *topology GP map* has all the neutral connections of the original NC, but randomized non-neutral mutational neighborhoods, thus erasing non-neutral correlations (similar to McCandlish 2012). The *community GP map* also randomizes non-neutral mutational neighborhoods but only performs swaps within a network community, thus only partially erasing non-neutral correlations. The rightmost drawing represents the full NC from the *RNA GP map*, and the three structures are shown next to it. To make the figure easier to interpret, only an excerpt is shown for the random GP map.

To investigate how different features of the RNA GP map can lead to overdispersion in the arrival of novel phenotypic variation, we construct a hierarchy of simpler models that contain increasing amounts of the structure of the full RNA data. These are depicted in Fig. 3 and discussed in more detail below.

At the first and simplest level, we use a model from Schaper and Louis (2014), the *random GP map*, which has discrete genotypes, but no correlations. The topology and genetic neutral and non-neutral correlations of the NC are completely erased by randomly assigning sequences of length L=12nt (the genotypes) to phenotypes (secondary structures) subject to the constraints that the mean outcomes of both neutral and non-neutral mutations equal those of the gray NC in the RNA map. In this map, not every genotype on the NC is a portal genotype, simply due to statistical fluctuations.

At the second level, we define a *topology GP map*. Here, the initial NC and its internal topology (all neutral mutation connections) are identical to the full RNA map, but the phenotypic changes generated through non-neutral mutations are randomized (similar to a model found in McCandlish 2012). The mean probability of a specific phenotypic change is set to match the corresponding NC in the RNA map, but *which* non-neutral mutation gives *which* phenotypic change is completely randomized. Thus, this map reproduces all neutral genetic correlations, but it will have no non-neutral genetic correlations.

At the third level, we define a *community GP map*. As for the topology GP map, the initial NC and its topology are identical to the full RNA map. Unlike the topology GP map, however, the randomization of non-neutral mutations is applied to each network community of the NC separately. Thus, this map captures the fact that some phenotypic changes might be more likely in certain network communities of the RNA NC but misses out on other kinds of non-neutral genetic correlations.

Finally, we investigate the full *RNA map*. In addition to the features present in the community GP map, we observe that non-neutral mutations to a specific phenotype are even more clustered to specific genotypes and their neighborhood.

### Overdispersion in Arrival Rates on the RNA GP Map

We start with population dynamics simulations of the case where all non-neutral variants are unviable (i.e. have zero fitness). Although alternative phenotypes are introduced through mutations, they then disappear within one generation due to the strong stabilizing selection. With this simplification, the population will be confined to the initial phenotype $p_{g}$^b^ and we can study the introduction of new variation in isolation, without any ongoing non-neutral fixation processes.

**Fig. 4.**
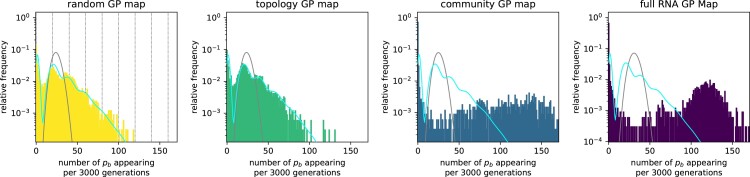
Strong deviations from Poisson statistics for the appearance of phenotype $p_{b}$ in a population neutrally evolving with stabilizing selection for $p_{g}$. Phenotype appearances are quantified by splitting the simulation into time intervals of Δt=3,000 generations and recording how often the given new phenotype $p_{b}$ appears in each Δt. These data are shown for all four GP map models. The number of appearances per interval is highly overdispersed compared to a Poisson distribution with the same mean (gray line), which would be expected from an average-rate model. For the random GP map, the data can be approximated analytically (cyan line, given by supplementary Equation (S13), Supplementary Material online). Vertical lines highlight the values $n_{p_{b}}\Delta t/t_{\mathrm{gene}}$ for a range of values $n_{p_{b}}$, depicting the expected number of $p_{b}$ mutants if a perfectly monomorphic population was located at a genotype with exactly $n_{p_{b}}$ discrete instances of $p_{b}$ in its mutational neighborhood. The data on the community GP map and full RNA GP map show even higher overdispersion than analytically predicted for the random map. Parameters: population size N=1,000, mutation rate $u=2\times 10^{-5}$, total time $10^{7}$ generations. The initial NC is the one shown in Fig. 3 and $p_{b}$ corresponds to the blue phenotype in the same figure. Many further examples for other phenotypes and RNA sequences of length L=30 nt can be found in supplementary Sections S2.1 and S2.4, Supplementary Material online.

To measure the statistics of the introduction of new mutations, we simulated a population of N=1,000 haploid individuals with a mutation rate per site of $u=2\times 10^{-5}$ using Wright–Fisher dynamics (see Methods). Since NLu≈0.24, this system is in the monomorphic regime. We record how many times one specific new phenotype, $p_{b}$, appears during each interval of Δt=3,000, which is much shorter than the neutral fixation time-scale of $t_{\mathrm{ne}}\approx 1.2\times 10^{4}$ generations. From these data, overdispersion can easily be observed as a deviation from a Poisson distribution (gray curve in Fig. 4). We find marked deviations from Poisson statistics for all four maps: intervals with zero appearances of $p_{b}$ and intervals with a very high number of $p_{b}$ appearances are much more common in the simulation data than for a Poisson distribution with the same mean. This is clearest for the full RNA map data, where only 0.18% of all time intervals have $p_{b}$ counts in the μ±σ (where *σ* is the standard deviation) range of the Poisson distribution: the counts in 73% of time intervals fall below this range (representing the time between bursts), while 27% appear above the range (the bursts). Similar findings hold for other phenotypes $p_{i}$ (supplementary Section S2.1, Supplementary Material online), as well as for longer sequences of length L=30 nt (supplementary Section S2.4, Supplementary Material online).

For the random GP map, the simplest of our GP map models, we can estimate the overdispersed distribution analytically, which provides a reasonably good fit to the data (cyan line in Fig. 4) by capturing the following simple phenomenology: if the population is perfectly monomorphic and remains on the same genotype $g_{0}$ throughout the time interval, then the expected number of $p_{b}$ mutants produced is simply given by the number of $p_{b}$ phenotypes in the mutational neighborhood, $n_{p_{b}}$, multiplied by the number of times each mutation occurs during Δt, which is given by $\Delta t/t_{\mathrm{gene}}$ (from Equation (2)). Thus, the expected number of $p_{b}$ appearances depends on the current prevalent genotype $g_{0}$ through $n_{p_{b}}$. Since $n_{p_{b}}$ can be any non-negative integer, we expect a weighted sum of Poisson distributions, one for each $n_{p_{b}}$: a peak at zero, and then successively smaller peaks at $\Delta t/t_{\mathrm{gene}}$, at $2\Delta t/t_{\mathrm{gene}}$, etc. These values are shown as black dotted lines in the random map histogram and are close to the peaks observed in the full distribution. The full analytic expression represented by the cyan line includes some further effects such as the fact that the population can fall off a portal genotype during Δt and that our populations are not perfectly monomorphic.

Having described the dynamics on the simple random map, let us compare all four GP maps. To help identify differences between the four distributions in Fig. 4, the cyan line that approximates the distribution for the random GP map is included in all four subplots. First, note that the distributions from the random GP map and the topology GP map are quite similar. This similarity is perhaps not surprising, because in each map the portal genotypes are uniformly distributed across the NC. Next, we note that the overdispersion increases for the community GP map and even more for the full RNA GP map. These maps have an inhomogeneous distribution of portal genotypes over the NC. Thus, a population will not produce $p_{b}$ when it is neutrally diffusing across areas of the NC that are depleted in portal genotypes for $p_{b}$, and will repeatedly find portals when it is in a region that is enriched in them, leading to further overdispersion. The community structure of the neutral network can reinforce this effect by slowing down the time-scale to go from one part of the NC without portals to one with portals (McCandlish 2012).

From these observations, we can deduce several factors that contribute to the overdispersion in phenotypic variation. First, having finite and discrete mutational neighborhoods is a sufficient condition for overdispersion, as predicted by the scaling arguments reviewed at the beginning of this paper and shown here for the random GP map. Second, the similarity of the data from the random GP map and the topology GP map indicates that the topology of the NC in itself, which is caused by neutral correlations, may not lead to much additional overdispersion in the production of novel variation. Third, the non-neutral genetic correlations that are present in the community GP map and the full RNA GP map can cause additional overdispersion. In the full RNA GP map, the distribution actually has a secondary peak at rates that are much higher than the mean. This extra peak is caused by the fact that it is no longer an exception to have several instances of $p_{b}$ in a mutational neighborhood since the few possible transitions to $p_{b}$ are grouped around a very small part of the NC, as can be seen in Fig. 3. While the strength of these non-neutral correlations will depend on many details of the GP map (Greenbury et al. 2016) and differ for different target phenotypes (McCandlish 2012), one simple source follows from the generic high robustness of all NCs (Greenbury et al. 2016; Mohanty et al. 2023): if a genotype that maps to the blue phenotype has several mutational neighbors that also map to the blue genotype, likely, a few of these are also mutationally accessible from one specific part of the gray NC.

### Influence of Mutation Rates and Population Sizes

So far, we have found overdispersion in the arrival rates of non-neutral phenotypic variation for several GP map models, but only considered a single population size *N* and mutation rate *u* in each case. Next, we vary these two parameters. For this analysis, we need a single number that summarizes how much the phenotypic variation found in a population deviates from a Poisson process. Here we consider the time intervals $t_{r}$ between two successive and non-concurrent appearances of $p_{r}$, which follow an exponential distribution in a Poisson process,^c^ and focus on the coefficient of variation, defined as


(7) $$V_{t_{r}}=\frac{\sigma_{t_{r}}}{\mu_{t_{r}}},$$

where $\mu_{t_{r}}$ and $\sigma_{t_{r}}$ are the average and standard deviation of the time interval distribution. For a Poisson process, we would have $V_{t_{r}}=1$ (Goh and Barabási 2008). Higher values of the coefficient of variation indicate an overdispersed scenario, as in Fig. 1b, where very short times between two $p_{r}$ appearances (within “bursts”) and very long times between $p_{r}$ appearances (between “bursts”) are common. Since there are limitations in quantifying the burstiness of finite data sets using simple metrics based on the coefficient of variation (see, for example, Kim and Jo 2016), we also provide the full data in the supplementary Section S2.2, Supplementary Material online.

**Fig. 5.**
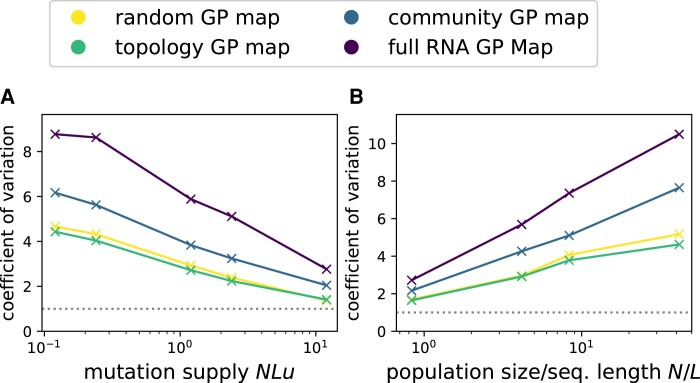
How does the amount of overdispersion, quantified by the coefficient of variation from Equation (7), depend on being in the monomorphic regime NuL≪1 or on being in the slow-drift regime N/L≫1? We repeat the simulations from Fig. 4: in a) we vary the mutation rate *u* at a constant population size N=200 to study the effect of leaving the monomorphic regime. In b) we vary the population size *N* at a constant mutation rate $u=5\times 10^{-5}$ to study the fast-drift regime. The initial and final phenotypes are the gray and red phenotypes in Fig. 1. Each line in the plot stands for a different GP map (see legend). The gray dashed line denotes the Poisson statistics prediction $V_{t}=1$. Since the number of (neutral and non-neutral) mutations per generation scales as NLu, we need longer run-times to obtain reliable statistics for lower values of NLu and thus run simulations for $T=\text{max}(10^{6}/(NuL),10^{4})$ generations, always rounded up to the nearest power of ten.

We simulate Wright–Fisher dynamics on all four maps for a range of mutation rates *u* and population sizes *N* and summarize the statistics by the coefficient of variation from Equation (7) in Fig. 5. We can draw the following conclusions from the coefficients of variation: first, in agreement with the previous section, the community and RNA GP maps display the most overdispersed dynamics, and the random GP map and the topology GP map are approximately similar.

Second, as can be seen in Fig. 5a, overdispersion is strongest when the population is in the monomorphic regime, and $V_{t_{r}}$ reduces as the population becomes more polymorphic with increasing NLu. The highest mutation rate in our data gives a highly polymorphic population with NuL=12, where any two individuals are expected to have incurred ≈24 mutations since their last common ancestor *N* generations ago (see Drossel 2001 with a total mutation rate of *uL*). For this population, the random and topology map data are near the Poisson statistics expectation of $V_{t_{r}}=1$, but the dynamics are still overdispersed for the full RNA GP map, suggesting that the population needs to be even more polymorphic before it spreads enough over the NC to wash out the larger-scale fluctuations in the distribution of portal genotypes illustrated in Fig. 1a.

Finally, as shown in Fig. 5b, the overdispersion becomes weaker in the fast-drift limit N/L<1. In this limit, the population will move to a new neutral phenotype before the genotypes in its 1-mutational neighborhood appear repeatedly to produce a burst.

To sum up, we find that overdispersion is strongest for large populations with low mutation rates, as expected from the simple scaling arguments from Schaper and Louis (2014) reviewed at the beginning of this paper. However, for the full RNA GP map, we observe overdispersion further into the polymorphic limit and the fast-drift limit with N/L<1 than the simple scaling arguments suggest. These observations generalize to further phenotypes (see supplementary Section S2.3, Supplementary Material online).

### How Bursts Affect Fixation Times

**Fig. 6.**
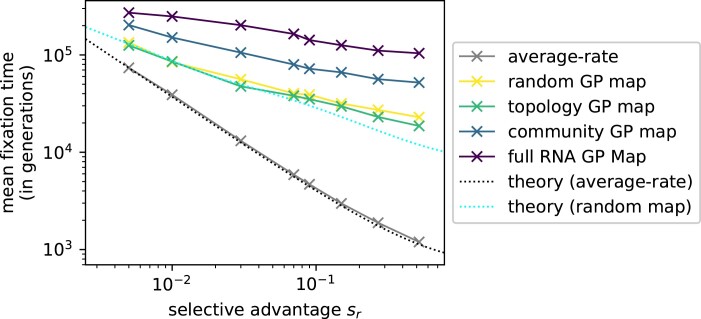
Overdispersion weakens the influence of the selective advantage $s_{r}$ of an adaptive phenotype on its time until fixation. The population starts on the NC of the initial phenotype $p_{g}$ and a single phenotype $p_{r}$ has a selective advantage of $s_{r}$ over $p_{g}$. All other phenotypic changes are deleterious with zero fitness. We repeat the simulation $10^{3}$ times for each value of $s_{r}$ and record how many generations it takes on average from the start of the simulation until $p_{r}$ fixes (thus, the time includes the introduction through mutations and fixation). Data are shown for all four GP maps (the random, topology, community, and RNA GP maps), as well as for an “average-rate” model, where variation is introduced by a random number generator at a fixed rate for each phenotype. In all cases, $p_{r}$ fixes more rapidly if its selective advantage is higher, but this decrease is much steeper for the average-rate model than for the GP map models, which have overdispersed variation. Classic origin-fixation theory (McCandlish and Stoltzfus 2014; gray line, Equation (8)) describes the “average-rate” simulations well. The flatter scaling on the GP map models is captured by a simple analytic approximation for the random GP map (cyan dotted line, supplementary Equation (S15), Supplementary Material online). Parameters: population size $N=10^{3}$, mutation rate $u=2\times 10^{-5}$ so that M≈78 for the random map, and NLu≈0.24. The initial NC is the same as in the preceding Figs. 3-5, and $p_{r}$ is the phenotype drawn in red in Fig. 1.

In this section, we will test our earlier scaling arguments about fixation. For simplicity, we consider a simple adaptive scenario, where only a single phenotype $p_{r}$ has a selective advantage over the initial phenotype $p_{g}$ and all remaining phenotypes are unviable. In this case, the outcome is clear: at some point, the fitter phenotype $p_{r}$ will go into fixation. Nevertheless, the timing of this fixation will depend on the timing of $p_{r}$ introductions and the strength of its selective advantage. The higher the selective advantage, the more likely an individual $p_{r}$ mutant is to go into fixation and so the fewer $p_{r}$ mutants are required for fixation and the lower the fixation time. As shown in Fig. 6, this negative correlation between fixation time and selective advantage is indeed observed in all four maps, as well as for an average-rate scenario. However, the decrease of fixation time is much greater in the average-rate case than in the GP maps: as the selective advantage $s_{r}$ increases by approximately two orders of magnitude in Fig. 6, the mean fixation time decreases by a factor of ≈62 in the average-rate simulations, compared to just ≈2.6 in the simulations on the RNA map. As discussed in our section on scaling arguments, this weak dependence on the selective advantage can be explained by the presence of bursts: once a selection coefficient is larger than a threshold that scales as 1/(burst size), the new phenotype will fix almost certainly during the first burst and so increasing the fitness further will only weakly affect fixation time.

To test our quantitative understanding of the dynamics, we also plot analytic approximations for the average-rate model and the random map in Fig. 6. For the average-rate case, the mean fixation time $t_{\mathrm{fix}}$ will scale inversely with the product of an average-rate origin term $r_{r}$ and single-mutant fixation probability $P_{\;p_{r}}^{\mathrm{fix}}$, like in a classic origin-fixation model (McCandlish and Stoltzfus 2014; details in supplementary Section S1.2.2, Supplementary Material online):


(8) $$t_{\mathrm{fix}}\approx (r_{r}\times P_{\;p_{r}}^{\mathrm{fix}}{)}^{-1}.$$

To match the GP map averages, the average-rate origin term should be $r_{r}=\phi_{\;p_{r}p_{g}}NuL$, i.e. the product of the mutation supply NuL and the mean probability $\phi_{\;p_{r}p_{g}}$ that a phenotype $p_{r}$ appears upon random mutations from that NC (Schaper and Louis 2014). The second term, the single-mutant fixation probability for Wright–Fisher dynamics, is given in Table 1. With this, we can estimate the time to fixation in the average-rate case (gray line in Fig. 6), which approximates the data from the computational average-rate model well.

For the random map, estimating the time to fixation of $p_{r}$ is more complex and thus derived in the supplementary Section S1.3.4, Supplementary Material online. The calculations are based on the scaling arguments from Equations (4) and (5), but with some further terms to approximate how the presence of some neutral genotypic variation in the populations leads to deviations from “perfect” bursts described by the scaling arguments. The prediction is shown in Fig. 6 and fits the data well for the random GP map and the topology GP map. Deviations between the curves only appear for highly adaptive phenotypes with high $s_{r}$, most likely because their fixation time is most sensitive to the neutral genotypic variation in the population (see supplementary Section S5.2, Supplementary Material online, for more details), which we only treat approximately.

To sum up, both our analytic calculations and simulation results indicate that the selective advantage of the adaptive phenotype $p_{r}$ has a (much) lower impact on its fixation time in the overdispersed scenario than in the average-rate model, as was predicted by the scaling arguments at the beginning of the paper, especially Equation (5).

### Implications of Overdispersion for Adaptation with Two Fitness Maxima

**Fig. 7.**
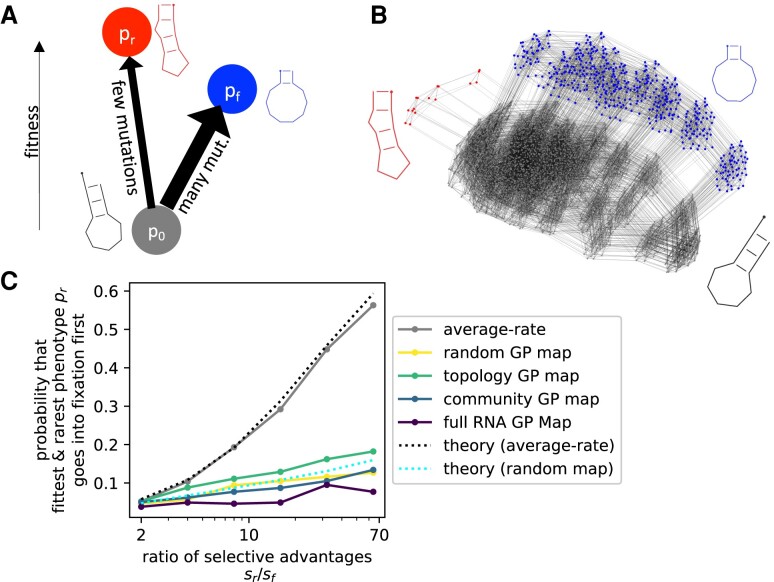
Overdispersion affects fixation probabilities in a landscape with two fitness maxima. a) Sketch of the fitness landscape (scenario from Schaper and Louis 2014). The population initially starts with phenotype $p_{0}$ and can evolve toward one of two local maxima, phenotype $p_{f}$ or $p_{r}$. $p_{r}$ is the global fitness maximum but is less likely to arise through mutations (thus *r* for mutationally *rare* with $\phi_{\;p_{r}p_{0}}\approx 2.3\times 10^{-4}$ and *f* for *frequent* with $\phi_{\;p_{\;f}p_{0}}\approx 7.4\times 10^{-3}$). b) Sketch of the full mutational network relevant to this fitness landscape. c) For each of the GP map models from Fig. 3, as well as for an “average-rate” model, where $p_{f}$ and $p_{r}$ are introduced with constant probabilities, we record the probability that the fitter, but mutationally rarer phenotype $p_{r}$ goes into fixation first. This probability is plotted against the selective bias toward $p_{r}$, i.e. the ratio of the selective advantages $s_{r}$ and $s_{f}$, both relative to $p_{0}$. In all cases, a higher selective bias toward $p_{r}$ makes it more likely for $p_{r}$ to fix, but this trend is less pronounced for the overdispersed dynamics on the GP maps. The simulation results are well predicted by theoretical calculations both for the random GP map (cyan dashed line, Equation (10)) and for the average-rate model (gray dashed line, Equation (9)). Parameters: N=500, $u=2\times 10^{-5}$, probabilities based on 1,000 repetitions.

In the previous section, the outcome was always clear: there was exactly one phenotype with a selective advantage and this phenotype went into fixation in all simulations. The only question was its timing. In this section, we investigate a more general case treated for example in Yampolsky and Stoltzfus (2001) and Schaper and Louis (2014), where two phenotypes, $p_{f}$ (for “frequent”) and $p_{r}$ (for “rare”), have selective advantages over the initial phenotype $p_{0}$ and either of them could go into fixation first. These two phenotypes, $p_{f}$ and $p_{r}$, have different mean likelihoods to appear through random mutations, $\phi_{\;p_{\;f}p_{0}}$ and $\phi_{\;p_{r}p_{0}}$, and they have different selective advantages, $s_{f}$ and $s_{r}$, over the initial phenotype $p_{0}$, as sketched in Fig. 7a. We are primarily interested in whether $p_{f}$ or $p_{r}$ will go into fixation first, as in Yampolsky and Stoltzfus (2001) and Schaper and Louis (2014). Therefore, we chose phenotypes $p_{f}$ and $p_{r}$ that are not connected by point mutations, such that both phenotypes constitute a local maximum that is difficult to escape from.

The most interesting scenario is when the less frequent phenotype $p_{r}$ has the higher selective advantage, such that the biases in variation and selection favor different phenotypes. For the average-rate model, the probability $P_{\mathrm{rfixes}}$ that the fittest phenotype $p_{r}$ is the first to fix is given by a very simple ratio in the origin-fixation regime (derived in supplementary Section S1.2.3, Supplementary Material online, equivalent to the classic result from Yampolsky and Stoltzfus 2001):


(9) $$P_{r\;fixes}={\left(1+\frac{P_{\;p_{f}}^{\mathrm{fix}}\phi_{p_{f}p_{0}}}{P_{p_{r}}^{\mathrm{fix}}\phi_{p_{r}p_{0}}}\right)}^{-1}\approx {\left(1+\frac{s_{f}\phi_{p_{f}p_{0}}}{s_{r}\phi_{p_{r}p_{0}}}\right)}^{-1},$$

where the second approximate step holds for $1/N\ll s_{i}\ll 1$. There is only one effective parameter that sets the probability of the final outcomes, namely the ratio of the two origin-fixation terms. This simple analytic prediction is shown as a gray dotted line in Fig. 7c and is in good agreement with our simulation results for the average-rate scenario. In other words, if we replace the GP structure of Fig. 7b with average rates, then Equation (9) works very well.

How do the four levels of GP map structure affect the probability of different outcomes? We observe in Fig. 7c that a higher selective advantage of $p_{r}$ still raises the probability that $p_{r}$ fixes, but this increase is dramatically weaker for the bursty dynamics on the GP maps. The reason follows from our arguments about fixation times: if we are in the saturating regime of large bursts with sM≫1, the outcome is set primarily by whether the first burst to appear is one of $p_{f}$ or $p_{r}$ mutants, which in turn is set by the probability of finding a portal genotype and not by selection. If we assume that $p_{r}$ and $p_{f}$ appear and fix independently from one another, then the probability that $p_{r}$ fixes first depends on their individual fixation times $t_{i}^{\mathrm{fix}}$ as follows (see supplementary Section S1.1.1, Supplementary Material online):


(10) $$P_{r\;\mathrm{fixes}}=(1+t_{r}^{\mathrm{fix}}/t_{\;f}^{\mathrm{fix}}{)}^{-1},$$

which reduces to Equation (9) if we use the average-rate expression (Equation (8)) for the fixation times. If instead we use the fixation times from our random GP map calculations (supplementary Equation (S15), Supplementary Material online), then we find good agreement with the simulations for the random map (see the teal line in Fig. 7). Thus, the reduced sensitivity to selective advantages in this calculation simply comes from the reduced sensitivity to selective advantages in the fixation times. Given the success of Equation (10) for the random GP map, we show in supplementary Section S1.1.2, Supplementary Material online, that Equation (10) can easily be generalized to multiple peaks, as long as we assume that the different phenotypes are introduced independently from one another. If there are *n* adaptive phenotypes, the probability that phenotype $p_{1}$, with fixation time $t_{1}^{\mathrm{fix}}$, will fix before the others is given by


(11) $$P_{{\text{p}}_{1}\mathrm{fixes}}={\left(1+t_{1}^{\mathrm{fix}}\sum_{i=2}^{n}\frac{1}{t_{n}^{\mathrm{fix}}}\right)}^{-1}.$$

We demonstrated via simulations that in the presence of bursts, the first fixation event on the two-peaked landscape can depend much less on the selective advantages of the peaks, and much more on how likely they are to appear as potential variation than one would expect from average-rate models. Note that this scenario differs from the “survival of the flattest” effect (Wilke et al. 2001; Tejero et al. 2011), which also predicts preferential fixation for phenotypes with larger neutral sets or higher robustness, but which only applies at high mutation rates. Similarly, arguments based on “free-fitness” (Iwasa 1988; Sella and Hirsh 2005) can also be used to explain why phenotypes with larger neutral sets are more likely to fix. The free-fitness formalism is inspired by statistical mechanics and depends on steady-state assumptions. It would be more appropriate for a fixed fitness landscape on much longer time scales when the population has repeatedly transitioned between the different phenotypes. In such a setting, the details of the short-term dynamics would be less important, including the effects of bursts. Nevertheless, because phenotypes with larger neutral sets tend to have shorter $t_{\mathrm{port}}$ (Schaper and Louis 2014), all of these different limits above end up predicting a relative preference for phenotypes with larger neutral sets. The question of precisely where in biology we should expect each scenario to hold remains open.

### Overdispersion in Arrival Rates on Alternative GP Maps

In this paper, we have primarily used the sequence-to-RNA-secondary-structure GP map to computationally analyze the causes and consequences of bursts. Many of our arguments for this specific system should hold more generally. We therefore study two more GP maps in this section.

**Fig. 8.**
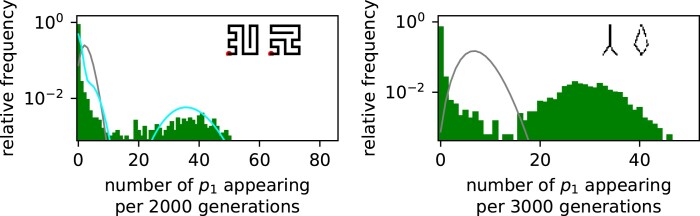
Strong deviations from Poisson statistics in two further GP maps, a protein lattice model and Richard Dawkins’s biomorphs. The analysis in Fig. 4 is repeated on two different GP maps: (left) the HP lattice model, a simple model of protein folding, where genotypes are mapped to phenotypes based on free-energy minimization. The initial and final phenotypes used in this specific analysis are shown in the top-right corner. (right) Richard Dawkins’s biomorphs, a toy model of development, where numeric phenotypes are mapped to 2D images as phenotypes based on a recursive growth process. The initial and final phenotypes used in this specific analysis are shown in the top-right corner. Data for further choices of phenotypes in each of these two maps are found in the supplementary Section S3, Supplementary Material online. Parameters: population size N=1,000, mutation rate $u=2\times 10^{-5}$, and total time $10^{7}$ generations.

First, we simulate an evolving population on a GP map defined by the HP lattice model, a simple and popular schematic model of protein folding (Dill 1985; Li et al. 1996). A genotype is any string of residues of type “H” (hydrophobic) and “P” (polar). A phenotype is defined as the minimum-free-energy lattice configuration of that genotype. This GP map is conceptually similar to that of RNA. In both cases, a genotype made up of letters from a fixed alphabet (“GACU” for RNA, “HP” in the protein lattice model) is mapped to a discrete folded structure based on biophysical rules. We observe from simulations that new phenotypes are introduced in an overdispersed fashion (Fig. 8, left), similar to what was found for RNA in Fig. 4. However, unlike for RNA, the observed distribution is not very different to that of the corresponding random map (drawn as a teal line), indicating that genetic correlations, which distinguish a GP map from its corresponding random map, may be weaker in this map, in agreement with prior direct measurements of the genetic correlations in this model (Greenbury et al. 2016).

As a second GP map, we turn to Richard Dawkins’s biomorphs, first proposed in “The Blind Watchmaker” (Dawkins 2016) as a toy model of how developmental processes bridge between the genotypic and phenotypic levels. In this model, genotypes made up of nine integers are fed into a recursive growth process to produce 2D drawings as “phenotypes”. Although this model differs substantially from the biophysical models discussed so far, we nevertheless still find overdispersed phenotypic introductions in Wright–Fisher simulations of a population evolving on this GP map (Fig. 8, right). Comparisons with our analytic calculations were not done for the biomorphs system since the numeric nature of the biomorphs’ genotypes would complicate our calculations too much.^d^

Taken together, these results show that the overdispersion in the introduction of new phenotypes which we found for the RNA GP map carries over to two further GP maps, one biophysical one mimicking protein folding and a more schematic one representing recursive growth processes.

## Conclusions and Discussion

### Main Conclusions

Non-Poissonian bursts of size *M* in the arrival of novel phenotypic variation *p* are typical in evolutionary models on GP maps, as long as some phenotypes are only accessible from a relatively small number of “portal” genotypes, and the population is in the weak-mutation or monomorphic regime (McCandlish 2012; Schaper and Louis 2014). Here we explore these bursts in detail, showing in particular that they strongly affect adaptive dynamics. In particular, above a threshold of selection coefficients s≳1/M≪1, the time to fixation is dominated by the probability of finding a portal genotype, and so depends only weakly on fitness.

To explore the effects of “bursty” statistics on the arrival and fixation of novel phenotypic variation, we focused on the RNA GP map. We measured the amount of overdispersion for a set of five models with increasing amounts of structure, which allowed us to investigate several different causes of overdispersion. Adding the full connectivity of the NC, e.g. including all neutral correlations, does not change the overdispersion by much beyond simply taking the discrete and finite nature of mutational neighborhoods into account. However, the introduction of further non-neutral genetic correlations, such as those quantified by Greenbury et al. (2016), can markedly increase the amount of overdispersion. Roughly speaking, these non-neutral correlations imply that genotypes that have access to a novel phenotype in their one-mutational neighborhood are clustered together in the NC, generating additional sources of bursty behavior. We further demonstrated that, as predicted in Schaper and Louis (2014), the amount of overdispersion in the introduction of a novel phenotype further depends on the details of the evolving population: genetically diverse populations or small populations undergoing rapid genetic drift average over several genotypes in the NC and therefore produce new phenotypes at a more constant rate than large monomorphic populations do.

By evolutionary dynamics simulations on the RNA GP map, we gave explicit examples of where overdispersion in the arrival of novel phenotypic variation impacts the dynamics of adaptation. We showed how bursts imply that fitness differences play a smaller role in determining fixation times and evolutionary outcomes than in a Poisson model with the same average mutation rates. This reduced influence of selective advantages can be understood from a simple argument: the number *x* of introductions of *p* that are needed for a successful fixation event strongly depends on the selective advantage. In average-rate models, the time until *x* mutants appear depends linearly on *x*, but in a bursty model, the time until *x* instances of *p* appear can be approximated by the time to the first burst, as long as the burst size is M≫x. Then the exact value of *x* and thus the selective advantage becomes less important. One consequence of these phenomena is that if multiple phenotypes are fitter than the one on which the population resides, then bursts imply that the relative effect of differences in selection coefficients is much less important than it would be in an average-rate model. Instead, the probability of the population moving to a portal genotype, which depends on the frequency of the relevant phenotype as well as on the distribution of portal genotypes, plays a more important role in what eventually fixes. We explicitly demonstrated this effect for a two-peaked landscape and showed how to extend our analytic calculations to multiple peaks. We hypothesize that these effects of overdispersion may help explain why frequencies of phenotypes found in nature can in some cases (such as RNA secondary structures, Dingle et al. 2015, 2022, and the topology of protein complexes, Johnston et al. 2022) follow biases in arrival rates over many orders of magnitude even in the presence of natural selection.

### Dependence of Overdispersion on the Population Genetic Regime

Let us first review the conditions on the population parameters under which bursts are expected: our analysis in Fig. 5 indicates that bursts appear whenever the population is monomorphic (NuL<1) and sufficiently large (N/L>1), in agreement with the scaling arguments reviewed at the beginning of the paper and Schaper and Louis (2014). Burstiness persists for a larger parameter range if there are non-neutral correlations in the underlying GP map, such as in the community and full RNA GP maps. This is because populations need to spread out further over the neutral set to escape local heterogeneities, something they can achieve either through genetic diversity at high NuL or through fast genetic drift at low *N*. Thus, we observe burstiness on the RNA GP map even when NuL≈a or N/L≈b up to some finite constants a>1 and b>1 that depend on the strength of the non-neutral genetic correlations.

The second condition that the population is sufficiently large for the bursts to appear (N/L≳b) is likely to be met in most realistic cases. The first condition, however, that the population is monomorphic (NuL≲a), is more restrictive, and unlikely to be met for microorganisms, where populations are typically large, especially not for RNA viruses, which additionally have high mutation rates (Wilke 2004). These cases would be better described by the infinite-population limit commonly studied for GP maps (van Nimwegen et al. 1999; Aguirre et al. 2009; Capitan et al. 2015), where populations spread over many genotypes in a NC and average over the inhomogeneities that would otherwise lead to bursts. For effective population sizes $N_{e}$ in vertebrates, on the other hand, Lynch has estimated (Lynch and Conery 2003) that $N_{e}u$ is typically $0.00027<N_{e}u<0.0010$. In that case, $N_{e}uL<1$ for any genes with L≤1,000. Of course, the evolutionary dynamics for these classes of organisms are generally more complex than the simple model we used here, so further work is needed to work out when and where the effect of bursts will be most prominent.

### Generalization to Other Molecular and Developmental Phenotypes

Let us next turn to the conditions on GP maps for bursty dynamics: the minimum criterion is that only a fraction of genotypes in a NC are portals to phenotype $p_{i}$. That this should be generically the case follows from fairly general scaling arguments, and also from the positive link between neutral set size and evolvability (Wagner 2008), where neutral exploration allows a larger number of novel phenotypes to be discovered than would be possible from a single genotype. Another way of thinking about this aspect is in terms of epistasis since if only a few genotypes are portals, then the effect of a mutation depends on the genotype to which it is applied, even within a neutral set (Weiß and Ahnert 2018). Not all kinds of epistasis would lead to bursts as the following simple example shows: if every genotype has one mutation to $p_{i}$, but this mutation is at different sites for different genotypes, this would not lead to bursts. Nevertheless, epistasis is common in GP or genotype-fitness relationships (De Visser et al. 2011; Schaper et al. 2012; Domingo et al. 2019; Bank 2022; Johnson et al. 2023). A more detailed investigation is needed to flesh out the links between epistasis, which is quite a broad concept, and the conditions for bursts, before drawing further conclusions. More direct evidence for the prerequisites for bursts comes from GP map studies that have shown that different genotypes in a neutral set have different non-neutral mutational neighborhoods. Examples exist both in molecular GP maps (such as RNA; Fontana and Schuster 1998b; Wagner 2008) and higher-order GP maps (such as in a model of neural development, Psujek and Beer 2008, and gene regulatory networks, Ciliberti et al. 2007). Moreover, non-neutral correlations, i.e. cases where these differences exceed those expected in the random GP map, have been observed in a range of molecular GP maps (for example, RNA, protein quaternary structure, and protein tertiary structure; Greenbury et al. 2016) and are likely to exist in further GP maps.

One additional limitation of our results is that we restricted mutations to single nucleotide substitutions. However, since non-neutral correlations have also been found when single nucleotide insertions and deletions are included (Martin and Ahnert 2021), our results should generalize to a broader range of mutations. This question also calls for further study.

### Overdispersion and “Soft” Sweeps

Since bursts lead to repeated introductions of the same phenotype, they are reminiscent of the discussion around “soft sweeps” (Karasov et al. 2010; Jensen 2014; Schrider and Kern 2017), fixation events in which several advantageous alleles sweep to fixation in parallel. Whenever soft sweeps originate from new mutations, several advantageous alleles with similar selective advantages must indeed be introduced in close succession (Jensen 2014), such that they sweep to fixation in parallel without out-competing each other. While non-neutral correlations could raise the likelihood that two substitutions from a given genotype have the same phenotype and thus the same fitness, the bursts discussed in this paper are unlikely to be directly linked to “soft sweeps” since they are predominantly relevant in the “weak-mutation-strong-selection” regime (NuL≲1), where mutations arise rarely and sweep to fixation before further mutations occur (McCandlish and Stoltzfus 2014). Thus, even the time between two mutants in a “burst” would be too long for a joint fixation process.

### Future Work

The strong effect of the GP map structure on the statistics of the introduction of novel phenotypic variation observed here raises many directions for future research. First, there is the question of the strength of non-neutral correlations that amplify bursts beyond the simpler arguments based on finite mutational neighborhoods. This can only be addressed with more detailed ways of quantifying these correlations in different GP maps and by using the results in further calculations. There is a large parameter space to explore, with different GP maps, different NCs, and of course parameters such as population size and mutation rate.

Second, we derived expressions for the relative fixation rates for a two-peaked landscape or a multi-peaked landscape with one key assumption: that the introduction processes of *different* phenotypes are independent of one another. This assumption would break down, for example, if the mutational connections to two or more phenotypes of interest were clustered around the same part of the NC. Future work should address such phenomena both analytically and computationally.

Third, we have worked with a GP map, where each genotype corresponds to a single phenotype. Further questions arise around GP maps that have a non-deterministic relationship between genotypes and phenotypes (García-Galindo et al. 2023). Similarly, the concepts should be applied to transcription factor binding landscapes, which are also more complex GP maps, where each genotype can bind to multiple transcription factors with a varying quantitative binding strength (Aguilar-Rodríguez et al. 2017). Since *L* is typically short in this case, bursts could play a role.

Furthermore, our analytic approximations only use an approximate treatment of mildly polymorphic populations. While this is sufficient to estimate slight deviations from a perfectly monomorphic population, future work should provide analytic approximations for populations on GP maps that fill the gap between the idealized cases of highly polymorphic populations, the infinite-population limit, and the weak-mutation-strong-selection/monomorphic limit. Similarly, future work could relax the simplifying assumptions used in our computational simulations, for example, by using a continuous-time Gillespie model as in McFarland et al. (2013) instead of the simpler Wright–Fisher model.

Moreover, more detailed evolutionary models of specific evolutionary processes should include a more detailed treatment of the mutation process. While simple mutational biases such as a transition/transversion bias are unlikely to lead to qualitative changes in our predictions, there are interesting parallels between bursts and clusters of identical mutations arising from premeiotic mutational events (Woodruff et al. 1996), which may amplify the effect of bursts.

Finally, the big question is how to observe these effects experimentally. Exactly the points of difference between our bursts and existing analyses of overdispersion in adaptive evolution are two factors that make our burst difficult to investigate experimentally: first and most importantly, our bursts are fundamentally a *phenotypic effect*, and second, they are visible in the timing of *newly introduced* variation and only indirectly affect the timing of fixations. Thus, the bursts described in this paper can only be directly observed if we have both *genotypic* and *phenotypic* information for an evolving population for the entire population, even mutations that have just been introduced. An additional difficulty is that “bursts” are most prominent in the weak-mutation regime, whereas many well-studied examples of adaptation are microbes, which are not well approximated by this regime. Thus, direct evidence for bursts may be difficult to obtain. Despite these difficulties, experimental tests of our predictions should be designed and performed, and indirect evidence may be possible through the bursts’ effect on fixation, i.e. analyses similar to Fig. 6.

## Methods

### RNA GP Map

For the RNA GP map, we folded all possible sequences of length L=12 nt with the ViennaRNA package (Lorenz et al. 2011; default parameters, version 2.4.14). We took each sequence’s folded structure as this genotype’s phenotype but considered sequences to be non-folding if the mfe criterion was met by two degenerate structures. NCs are constructed in NetworkX (Hagberg et al. 2008) and drawn with its force-layout algorithm.

### Lattice Protein GP Map

For the lattice protein GP map, we use the code and parameters of Martin and Ahnert (2023): a compact lattice of size 5×5 for computational feasibility and an established energy model (Irbäck and Troein 2002), which simply favors hydrophobic–hydrophobic contacts by assigning them one free-energy unit.

### Biomorphs GP Map

Richard Dawkins proposed the biomorphs system, a simple recursive growth process, to illustrate how mutations on the genotypic level and selection on the phenotypic level can lead to successful adaptation (Dawkins 2016). In Dawkins’s original formulation, genotypes consisting of nine integers are mapped to two-dimensional images as phenotypes. For our computational analysis, we need to convert these images into discrete phenotypes, and for this, we use the data and code from our earlier work (Martin et al. 2024), where images were simply converted into binary pixels on a 30×30 grid. Unlike for the molecular models, the sequence length is fixed in the biomorphs, but the range of integers needs to be restricted to make genotype space finite and allow for computational analysis. As in our earlier work (Martin et al. 2024), we include all $\approx 4.6\times 10^{7}$ genotypes with $-3\leq g_{i}\leq 3$ for the first eight genotype positions and $1\leq g_{9}\leq 8$ for the ninth genotype position (the last position needs to be positive since it sets the number of recursions rather than being used for x/y-coordinates).

### Hierarchy of GP Maps

We start by identifying all sequences that belong to a given NC. Then we choose one initial NC in the RNA GP map to build simpler models for this NC (this was done twice, once for the NC in Fig. 1 and once for the one in Fig. 7). First, we determine the mean mutation rates $\phi_{\;p_{i}p_{0}}$ for that NC, i.e. what fraction of mutations starting at this NC give a specific new phenotype $p_{i}$. Note that all $\phi_{\;p_{i}p_{0}}$ sum to one by definition when the probability of neutral mutations, the robustness $\rho =\phi_{\;p_{0}p_{0}}$, is included. We also identify the network communities of this NC following Weiß and Ahnert’s (2020) method. Then the three simplified models were constructed from this NC information.

**Random GP Map:** In this map, phenotypes are allocated to genotypes at random and the only input are the frequencies of each phenotype (Schaper and Louis 2014). Here, we set the frequency of phenotype $p_{i}$ to $\phi_{\;p_{i}p_{0}}$, so that the mean mutation probabilities will match those for the initial NC in the RNA map.

**Topology GP Map:** Here the genotypes that form part of the initial NC are left unchanged so that the topology of this NC matches the one in the RNA map. The unchanged NC topology already ensures that the fraction of neutral mutations matches that in the RNA map. All remaining genotypes are assigned random phenotypes (except the initial phenotype $p_{0}$), each with a probability proportional to the rate from the RNA NC, $\phi_{\;p_{i}p_{0}}$. Here these probabilities had to be renormalized so all $\phi_{\;p_{i}p_{0}}$ without the neutral mutations for $p_{0}$ sum to one.

**Community GP Map:** Here, we start with the full RNA data and randomize the mutational neighborhood of one community in the initial NC at a time: for each community, we identify all genotypes that are mutational neighbors to this community, but not to another community in the initial NC. We shuffle the phenotypes associated with these genotypes to randomize the non-neutral mutations within each community. To keep the mean mutation probabilities intact, we identify subsets of genotypes with exactly *n* connections to the NC and only perform swaps within each subset.

### Simulations of Evolving Populations

For the evolutionary dynamics simulations on GP maps, we followed previous studies of evolutionary simulations on GP maps (Cowperthwaite et al. 2008; Schaper and Louis 2014) and implemented a Wright–Fisher model of a fixed number *N* haploid individuals in Python. Mutations were modeled to occur with constant probability *u* per reproduction event and site and the phenotype of the mutated sequence was given by the GP map. The population was initialized on a single genotype in the selected NC and then evolved neutrally for 10N generations before any data were collected, to randomize these forced initial conditions, as in Schaper and Louis (2014). We considered a fixation event to have occurred if less than 25% of the population carried the initial phenotype. To exclude rare and irreproducible jumps to other NCs of the neutral set of the initial phenotype $p_{0}$, which could confound our analysis, we set only genotypes in the initial NC of $p_{0}$ to fitness $f_{\;p_{0}}=1$ in all simulations on the *L* = 12 RNA, community and topology maps.

To simulate the average-rate scenario, we also performed simpler simulations without a GP map. Here, we simply assumed that L×u mutations occur per individual and generation, to match the GP map case, where the mutation rate is given per site. In the average-rate model, each mutation has the same probability $\phi_{\;p_{i}p_{0}}$ of giving phenotype $p_{i}$ and a constant probability $\rho =\phi_{\;p_{0}p_{0}}$ of leaving the initial structure unchanged (Schaper and Louis 2014). These rates are free parameters in the average-rate model, which we set to match the corresponding GP map values for the initial NC. For the rare event that a phenotype $p_{j}$ different from the initial phenotype exists in the population and mutates, we simply set rates that match the mean rates for mutations on that phenotype in the RNA map (rather than a specific NC of that phenotype).

## Supplementary Material

msae085_Supplementary_Data

## Data Availability

The code behind this analysis can be found at https://github.com/noramartin/evolutionary_dynamics

## Notes

a For RNA, the number of topologically possible structures scales as $\alpha^{L}\times L^{-3/2}$ with α≈2.29 (Hofacker et al. 1998), and the number of designable structures, i.e. structures that exist as phenotypes for at least one genotype, is also estimated to scale exponentially, as $0.13\times 1.76^{L}$ (Dingle et al. 2015).

b We also confine the population to a single NC, not the entire neutral set of the initial phenotype. This restriction ensures comparability between our hierarchy of GP map models by preventing rare cases where the population moves to a different NC from the neutral set of $p_{g}$ after a combination of a specific double mutation and genetic drift.

c Strictly speaking, a geometric distribution since time is measured in discrete generations.

d In the biomorphs GP map, each site is occupied not by a discrete letter representing a base, but by an integer, which can change by ±1 in a single mutation. Since we can only consider a finite number of genotypes, we have to set limits on the values that these integers can take and thus exclude some mutations (see Martin et al. 2024 for more details). This means that the number of allowed mutations per genotype (i.e. the size of a mutational neighborhood) varies from genotype to genotype, which is not considered in our analytic calculations and is probably an artifact of this particular model.
