# Supplementary material for: Non-Poissonian Bursts in the Arrival of Phenotypic Variation Can Strongly Affect the Dynamics of Adaptation
Source: Mol Biol Evol. 2024 May 2;41(6):msae085. doi: 10.1093/molbev/msae085 (PMC11156200; doi:10.1093/molbev/msae085)
Supplement: msae085_Supplementary_Data [file msae085_supplementary_data.pdf]

Electronic supplementary material: Non-Poissonian bursts in the arrival of phenotypic variation can strongly affect the dynamics of adaptation

Nora S. Martin, Steffen Schaper, Chico Q. Camargo, Ard A. Louis

Contents

S1 Analytic calculations **2**
S1.1 Standard results on statistical processes 2
S1.1.1 Two exponential processes: which occurs first 2
S1.1.2 Generalisation to more than two exponential processes: which occurs first 2
S1.1.3 Two exponential processes: when do we expect the first event 3
S1.2 Analytic calculations for the average-rate model 3
S1.2.1 Poisson distribution of appearance events (grey line in Fig 4 in the main text) 3
S1.2.2 Fixation times as a function of s_r (grey line in Fig 6 in the main text) . . 3
S1.2.3 Fixation probability of two competing mutants (grey line in Fig 7 in the main text) 3
S1.3 Analytic calculations for a simple overdispersed case: the random GP map 4
S1.3.1 Modelling mildly polymorphic populations 4
S1.3.2 Distribution of appearance events over a time interval (Fig. 4 in the main text) 5
S1.3.3 Fixation from a portal genotype 6
S1.3.4 Fixation times (Fig 6 in the main text) 7
S1.3.5 Fixation probability of two competing mutants (Fig 7 in the main text) . 8

S2 Additional data on overdispersed variation in RNA **8**
S2.1 Existence of bursts for a range of target phenotypes 8
S2.2 Dependence of burstiness on population parameters - more detailed plots 9
S2.3 Dependence of burstiness on population parameters for a range of initial conditions 12
S2.4 Longer sequence length of L = 30 nt 18
S2.5 Theoretical predictions of fixation times for sequence lengths L=50 nt and L=500 nt 20

S3 Overdispersed variation in other GP maps **21**
S3.1 Overdispersed variation in a lattice model for protein tertiary structure 21
S3.2 Overdispersed variation in Richard Dawkins's biomorphs, a toy model of development 22

S4 Effect of quasi-simultaneous p_1 mutants during a burst **27**
S4.1 Analytic treatment using the diffusion approximation 27
S4.2 Simulations with concurrent or individual mutants 28

S5 Impact of our simplified treatment of polymorphism **28**
S5.1 Amount of polymorphism - theory versus simulation 28
S5.2 Impact of polymorphism terms in our calculations 29

Symbol	Definition
f_0	fraction of the population at the most prevalent genotype g_0 in our approximation of mildly polymorphic populations (derived in Eq. S11)

Table 1: Symbols used in the calculations in addition to those defined in table I in the main text.

S1 Analytic calculations

In this section, we will derive an analytic expression for the expected rates, fixation times and fixation probabilities, first for the average-rate model from ref [17] and then for the overdispersed dynamics on the random GP map. In both cases, we can treat the system using a simple statistical treatment. If we wanted to model the full RNA map, we would need a more detailed treatment that explicitly includes the non-random, inhomogeneous structure of the GP map, such as the matrix treatment by McCandlish [15]. To simplify calculations, we ignore the time required for an ultimately successful mutant to actually go into fixation. All key variables for the calculations are summarised in table I in the main text.

S1.1 Standard results on statistical processes

Before we present analytic calculations for the average-rate model and the random map, let us start by reviewing three simple standard results on stochastic processes, which we will rely on repeatedly in our calculations.

S1.1.1 Two exponential processes: which occurs first

In the main text, we needed the probability that an event of type 2 occurs before the first event of type 1. Let us assume that the two events 1 and 2 occur at constant rates, one at rate $1/\tau_1$ and the other at rate $1/\tau_2$. Then the probability that the next event of type 2 occurs before the next event of type 1, i.e. $t_2 \leq t_1$, is given by an integral over the corresponding exponential process (if we neglect the fact that time is measured in discrete generations):

$$P = \int_0^{\inf} \frac{1}{\tau_1} e^{-\frac{t_1}{\tau_1}} \int_0^{t_1} \frac{1}{\tau_2} e^{-\frac{t_2}{\tau_2}} dt_1 dt_2 \tag{S1}$$

$$= \frac{1}{1 + \dfrac{\tau_2}{\tau_1}} \tag{S2}$$

S1.1.2 Generalisation to more than two exponential processes: which occurs first

Let us generalise the calculation in the previous section to more than three types of events (for example the fixation of three different phenotypes): if we assume that all three events can be described as three independent exponential processes, then we need to calculate the probability that the next event of type 1 occurs before the next event of types 2 and 3, i.e. $t_1 \leq min(t_2, t_3)$. We can simplify this calculation by combining types 2 and 3, which occur at rates $1/\tau_2$ and $1/\tau_3$ respectively, into a single exponential process with rate $(1/\tau_2 + 1/\tau_3)$ and time scale $(1/\tau_2 + 1/\tau_3)^{-1}$. Then we can use Eq. S2 to write down the probability that an event of type 1 occurs before the first event of the combined 2 & 3 process as:

$$P = \frac{1}{1 + \tau_1 \, (\frac{1}{\tau_2} + \frac{1}{\tau_3})} \tag{S3}$$

$$= \frac{1}{1 + \frac{\tau_1}{\tau_2} + \frac{\tau_1}{\tau_3}} \tag{S4}$$

This expression can easily be generalised to n outcomes (for example if instead of having a two-peaked landscape, we have an n-peaked one). In this case, the rate of all events except type one is $\sum_{i=2}^{n} 1/\tau_n$, which means that the probability that an event of type 1 is observed first is given by:

$$P = \frac{1}{1 + \tau_1 \left(\sum_{i=2}^{n} 1/\tau_n\right)} \tag{S5}$$

Note, however, that all this only holds while events can be approximated as independent exponential processes. This is not the case, for example, if τ_n stand for the appearance times of mutant n and several types of mutants are accessible from the same part of the NC.

S1.1.3 Two exponential processes: when do we expect the first event

Let us again assume that two types of events occur at constant rates, one at rate $1/\tau_1$ and the other at rate $1/\tau_2$. Then the total rate is given by

$$1/\tau_{tot} = 1/\tau_1 + 1/\tau_2 \tag{S6}$$

Therefore, the expected time until an event of *any* type is observed is given by:

$$\tau_{tot} = (1/\tau_1 + 1/\tau_2)^{-1} \tag{S7}$$

$$= \frac{\tau_1 \tau_2}{\tau_1 + \tau_2} \tag{S8}$$

S1.2 Analytic calculations for the average-rate model

Modelling mutations to p_1 as a simple Poisson process, i.e. occurring at a constant rate, as done e.g. in refs [2, 17], is the simplest approach. When selection also plays a role, we assume the origin-fixation regime, in which mutants are rare enough that each fixation process can be treated as an independent event and thus fixation probabilities can be written a simple product of mutation rates and fixation probabilities [16].

S1.2.1 Poisson distribution of appearance events (grey line in Fig 4 in the main text)

In the average-rate case, the number of p_1 mutants per time interval is a Poisson distribution. This distribution only has a single free parameter, the mean. In Fig 4 in the main text, we approximate the mean by the mean number of p_1 mutants per time interval in the corresponding GP map simulation.

S1.2.2 Fixation times as a function of s_r (grey line in Fig 6 in the main text)

p_r mutants are introduced at rate $r_r = LNu \times \phi_{p_r p_0}$ in the constant-rate model (ref [17] and table I in the main text) and the single-mutant-fixation probability is given by $P^{\mathrm{fix}}_{\mathrm{single}\ p_r}$ (see table I in the main text). Thus, if we ignore the time of the fixation process itself, and focus on the expected time until a p_r appears that will trigger a successful fixation event, we have:

$$t_r^{\mathrm{fix}} = 1/(r_r P^{\mathrm{fix}}_{\mathrm{single}\ p_r}) \tag{S9}$$

S1.2.3 Fixation probability of two competing mutants (grey line in Fig 7 in the main text)

In the origin-fixation regime, we can assume that all fixation events are independent and thus we model the fixation of p_f and p_r as independent processes occurring at constant rate with time

scales t_f^{fix} and t_r^{fix}, which are given by Eq. S9. Then it can be shown (details in section S1.1.1 of the *SI*) that the probability that p_r fixes before p_f is given by $P_{\text{r fixes}} = (1 + t_r^{\text{fix}}/t_f^{\text{fix}})^{-1}$.

In the limit $s_f \ll 1$ & $s_r \ll 1$ & $s_f N \gg 1$ & $s_r N \gg 1$, we have $P^{\text{fix}}_{\text{single } p_r} \approx s_r$, and so the expression can be rewritten as $P_{\text{r fixes}} \approx (1 + vf)^{-1}$, where v is the bias in variation, which is $v = \phi_{p_f p_0}/\phi_{p_r p_0}$, and $f = s_f/s_r$ the bias in selective advantages. This form agrees with Yampolski and Stoltzfus' [20] well-known result (after renormalisation since they compute the ratio $P_{\text{f fixes}}/P_{\text{r fixes}}$).

S1.3 Analytic calculations for a simple overdispersed case: the random GP map

The key aspect to model in the random map is the following: a population can move to a new genotype through neutral drift without changing its phenotype, but the new genotype can have different phenotypes in its mutational neighbourhood. In the random GP map, such differences in the mutational neighbourhoods are simple random fluctuations and can thus be modelled mathematically. Therefore, we can make analytic estimates for this case, relying on existing work in ref [17], where the random map model was first proposed.

S1.3.1 Modelling mildly polymorphic populations

As soon as we model evolution on a GP map, we need to account for the fact that a population of individuals with phenotype p_g can be made up of a mix of genotypes from the relevant NC of p_g. To keep our calculations simple, we will assume that one genotype, which we will call the 'prevalent genotype' g_0, dominates the population at any time. In other words, we are treating cases close to the monomorphic limit. Then we will approximate the population dynamics based on a single quantity, the prevalent genotype's frequency in the population f_0. In this approximation, the probability F that two individuals in the population carry the same genotype, a quantity closely related to the homozygosity [6], is well-approximated by the probability that two individuals carry g_0, i.e. we have $F = \sum_i f_i^2 \approx f_0^2$ (since the frequency of any other genotype f_i is low enough for f_i^2 to be negligible).

We can thus estimate f_0 if we know F, which we can approximate with coalescents (applying classic arguments [9] to the specific case of a NC on a GP map): the probability that two individuals in a population of size N have a common ancestor in the previous generation is $1/N(1 - uL(1 - \rho))$ (since the next generation is chosen from the $N(1 - uL(1 - \rho))$ individuals without deleterious mutations), and so on average the last common ancestor of two individuals is $N(1 - uL(1 - \rho))$ generations ago. Since this common ancestor, neutral mutations accumulate at rate $2uL\rho$ (the factor of two is due to the fact that the mutation can occur on either branch). Using again the expression for two exponential processes (section S1.1.1 of the *SI*), the probability that no neutral mutation has taken place in either of the two lineages since the common ancestor is approximately:

$$F \approx \frac{1}{1 + N(1 - uL(1 - \rho))2uL\rho} \tag{S10}$$

Using our previous approximation $F \approx f_0^2$, we can approximate the frequency of the prevalent genotype, f_0, as:

$$f_0 \approx \sqrt{\frac{1}{1 + N(1 - uL(1 - \rho))2uL\rho}} \tag{S11}$$

Note that we made several assumptions in this derivation, so it can only serve as a first approximation. The approximation is likely to get worse in the highly polymorphic limit ($NuL \gg 1$), but it has the correct limiting behaviour in the monomorphic limit ($f_0 \to 1$ as $NuL \to 0$) and it is found to be a sufficiently good approximation for the mildly polymorphic limit we encounter for the values of u, L and N that are used in this paper (see section S5.1).

S1.3.2 Distribution of appearance events over a time interval (Fig. 4 in the main text)

To model, how many times we expect a phenotype p_b to appear through random mutations, we will need to model how many instances of p_b exist in the mutational neighbourhood of the current prevalent genotype. In the random GP map, differences between the mutational neighbourhoods of different genotypes are caused by simple statistical fluctuations around a well-defined mean fraction of $\phi_{p_b p_g}$. Therefore, a binomial distribution can be used to describe the probability of finding n_{p_b} instances of p_b among the $(K-1)L$ possible mutations in the mutational neighbourhood of a genotype g_0:

$$P_{g_0}(n = n_{p_b}) = \binom{(K-1)L}{n_{p_b}} \phi_{p_b p_g}^{n_{p_b}} (1 - \phi_{p_b p_g})^{(K-1)L - n_{p_b}} \tag{S12}$$

Note that, for computational reasons, we used a phenotype with a relatively high $\phi_{p_b p_g} = 0.035$ in Fig. 4 in the main text. Then the probability of having portals with, for example, 1, 2 or 3 mutational neighbours mapping to phenotype p_b is given by $0.36, 0.23, 0.09$ respectively.

Next, we need to calculate how many p_b mutants we expect over a time interval Δt, given that we start with a prevalent genotype g_0 that has n_{p_b} instances of p_b in its mutational neighbourhood. Here, we simply assume that p_b mutants arise at a constant rate over a time scale Δt - but unlike the average-rate model, this rate itself depends on the current genotype and its value of n_{p_b}. However, our calculations also need to account for the fact that the prevalent genotype may change during Δt, and so we will sum over the probabilities of two cases:

- If the prevalent genotype in the population changes (i.e. a neutral fixation occurs) in the first half of the time interval, which occurs with $P(\text{neutral fixation middle}) = 1 - \exp(-0.5\ \Delta t / t_{ne})$: then the population averages over the mutational neighbourhoods of two genotypes, and we simply approximate this scenario with the average-rate model (see table I in the main text), so we expect $r_b \Delta t$ mutations of type p_b. It is important to note that this is a very approximate treatment, and thus only appropriate if neutral fixations within Δt are rare, i.e. $\Delta t \ll t_{ne}$.

- Otherwise, we assume that the prevalent genotype g_0 remains the same during Δt: since each genotype in the mutational neighbourhood is produced every t_{gene} generations, and there are n_{p_b} genotypes with p_b, p_b is produced at a rate n_{p_b}/t_{gene}. This rate applies only to the well-localised fraction f_0 of the population; let us assume that the average-rate model provides a good approximation for the remainder of the population. Thus, in this scenario the number of p_b mutations expected in Δt is $f_0 \Delta t n_{p_b}/t_{gene} + (1 - f_0) r_r \Delta t$.

Given the information on the rate at which we expect p_b to arise during a time interval Δt, the probability of finding exactly M mutants can be calculated using a Poisson distribution $\text{Pois}(M, x)$ with mean x. This gives:

$$\begin{aligned} &P(M \text{ mutants of } p_b \text{ in } \Delta t) = P(\text{neutral fixation middle}) \times \text{Pois}(M, r_b \Delta t) \\ &+ (1 - P(\text{neutral fixation middle})) \times \sum_{n_{p_b}=0}^{(K-1)L} P_{g_0}(n = n_{p_b})\ \text{Pois}(M, f_0 \Delta t n_{p_b}/t_{gene} + (1 - f_0) r_r \Delta t) \end{aligned} \tag{S13}$$

Here, the first term is reminiscent of the classic average-rate expression, which would be $\text{Pois}(M, r_b \Delta t)$ and corresponds to cases, where genetic drift smooths over differences between genotypes in the NC. The second term gives rise to the overdispersed behaviour: essentially fluctuations in p_b for different genotypes are described by the probability distribution $P_{g_0}(n = n_{p_b})$ and these fluctuations then give rise to large rate variations in the Poisson distribution.

S1.3.3 Fixation from a portal genotype

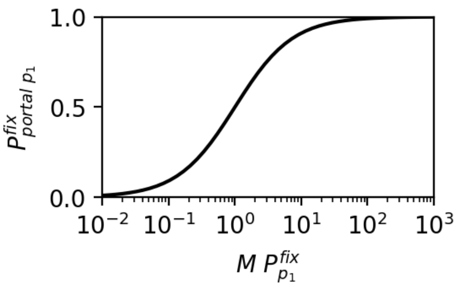

Figure S1: ***Probability of successful fixation event after a single burst on the random GP map*** $P^{fix}_{portal\ p_1}$***, as a function of the typical burst size*** M ***and the single-mutant fixation probability*** $P^{fix}_{p_1}$***:*** *for large enough burst sizes* M*, the function has saturated and the probability of a successful fixation within that burst is close to one, regardless of the exact value of the product* $MP^{fix}_{portal\ p_1}$*. This means that the outcome is less sensitive to small changes in the single-mutant fixation probability* $P^{fix}_{p_1}$*, which in turn depends on the selective advantage.*

A 'portal' genotype is any genotype with p_1 in its mutational neighbourhood, i.e. $n_{p_1} > 0$. Let us assume that a population is currently located at a portal genotype, which has exactly one instance of p_1 in its mutational neighbourhood (i.e. one out of $(K-1)L$ mutations from the portal genotype gives p_1). Then the population experiences a burst until the portal genotype changes through genetic drift. Let us compute the probability that p_1 will fix during that burst, i.e. before the portal genotype is lost through genetic drift on the neutral network. We can simply treat the two outcomes, a fixation to p_1 or a neutral fixation on the network, as independent, exponentially distributed random processes and compute the likelihood that the p_1 fixation occurs first. The timescales of these two random processes are:

- **Timescale of p_1 fixation:** p_1 appears every t_{gene} generations while the population is at the portal. The probability that a particular p_1 mutant goes into fixation is given by the single-mutant fixation probability $P^{\text{fix}}_{p_1}$ (table I in the main text). Then the time to p_1 fixation is therefore approximately $t_{p_1\ \text{fixes from portal}} = t_{gene}/P^{\text{fix}}_{p_1}$.
- **Timescale of neutral fixation:** t_{ne} (see table I in the main text).

Outside the strict monomorphic regime, and under the assumption that the fitter phenotype is only accessible from the portal genotype, the calculation only applies to a fraction f_0 of the population size, so we simply need to replace the population size N by Nf_0 when computing t_{gene}.

Thus, we can use the expression for the ordering of two exponential random processes (SI section S1.1.1) to compute the probability that p_1 fixes before a neutral fixation removes the portal genotype from the population:

$$\begin{aligned} P^{\text{fix}}_{\text{portal}\ p_1} &= (1 + t_{p_1\ \text{fixes from portal}}/t_{ne})^{-1} \\ &= [1 + \frac{t_{gene}}{t_{ne}P^{\text{fix}}_{p_1}}]^{-1} \\ &= [1 + \frac{1}{MP^{\text{fix}}_{p_1}}]^{-1} \end{aligned} \tag{S14}$$

In the last line, we have written this expression in terms of the burst size M.

This function is sketched in Fig S1 and we can distinguish two limiting cases:

1. If $P^{\text{fix}}_{p_1} M \gg 1$, then we have $P^{\text{fix}}_{\text{portal } p_1} \approx 1$. In this limit, a typical burst contains more than enough mutants for a successful fixation event. On the order of $1/P^{\text{fix}}_{p_1}$ mutants are needed for the phenotype to have a 50% probability of fixation, so if M is much larger, then the probability of fixation is large, and will remain insensitive to small changes in the selective advantage, as long as $P^{\text{fix}}_{p_1} M$ remains much greater than one. The time scale for fixation will therefore be dominated by the time to the next burst, rather than by $P^{\text{fix}}_{p_1}$.

2. If $P^{\text{fix}}_{p_1} M \ll 1$, then $P^{\text{fix}}_{\text{portal } p_1} \approx P^{\text{fix}}_{p_1} M$. Then the fixation probability from a portal phenotype is proportional to the single-mutant fixation probability, as before in the standard average-rate origin-fixation treatment in section S1.2.2.

S1.3.4 Fixation times (Fig 6 in the main text)

There are two ways in which a phenotype p_1 can fix: the first scenario is that the prevalent genotype in the population is a portal genotype to p_1. In this case, p_1 will arise repeatedly in quick succession and could fix. The second scenario is that a portal genotype exists among the wider set of less frequent genotypes in the population. Since the number of individuals with any particular genotype other than g_0 is expected to be small, we do not expect p_1 to arise repeatedly in the latter case. Thus, the first scenario is rarer (since it depends on having a portal as the prevalent genotype), but likely to lead to fixation when it occurs, whereas the second scenario is more common but less likely to lead to fixation. We will thus compute and then combine the fixation time scales for both scenarios:

- **Time scale t_p for fixation from a portal genotype:** similar to origin-fixation calculations [16], this expression is a product of a 'mutation term' (how often a portal genotype appears) and a 'fixation term' (how likely p_1 goes into fixation from a portal) - but unlike the average-rate equivalent, each term summarises an entire 'burst' of mutants (i.e. the period of time spent at a portal), rather than a single mutation.
  - How often does the population localise on a portal genotype through neutral drift? The time until the population localises on a portal genotype for a rare phenotype ($\phi_{p_1p_0} \ll 1$) can be approximated as follows (see also ref [17]): each genotype has $L(K-1)$ mutational connections and one in $\phi_{p_1p_0}^{-1}$ mutations from the NC give p_1. Then, on average one in $\phi_{p_1p_0} L(K-1)$ genotypes on the NC is a portal genotype to p_1. Since the prevalent genotype changes every t_{ne} mutations, the population will localise on a portal genotype approximately every $t_{ne}/(\phi_{p_1p_0} L(K-1))$ generations.
  - How likely is p_1 to go into fixation from a portal? This is given by $P^{\text{fix}}_{\text{portal } p_1}$ in Eq. S14.
- **Time scale t_c for fixation from a wider set of genotypes:** The part of the population that is not localised on g_0 is likely to contain a diverse set of genotypes, so we use an average-rate treatment, where the fixation time scale is given by Eq. S9. We only need to correct the average-rate mutation term for the fact that this treatment only applies to a fraction $(1 - f_0)$ of the population. This gives $t_c = (r_1(1-f_0)P^{\text{fix}}_{p_1})^{-1}$

Thus, we have two different mechanisms, by which p_1 could fix: either from a portal genotype on a time scale t_p or from a wider set of genotypes on a time scale t_c. The time scale at which at least one of these mechanisms has taken place and p_1 has gone into fixation if we assume the two mechanisms to be independent is $t_1^{\text{fix}} \approx (t_c^{-1} + t_p^{-1})^{-1}$ (see section S1.1.3). Putting this all together gives:

$$t_1^{\text{fix}} \approx (r_1(1-f_0)P^{\text{fix}}_{p_1} + \frac{\phi_{p_1p_0} L(K-1) P^{\text{fix}}_{\text{portal } p_1}}{t_{ne}})^{-1} \quad \text{(S15)}$$

We can understand this expression if we study its limiting behaviour:

1. If $t_c \ll t_p$, then most fixations take place from the wider cloud of individuals that do not carry the prevalent genotype. Then we have a fixation time of

$$t_1^{\text{fix}} \approx t_c = (r_1(1-f_0))^{-1}/P_{p_1}^{\text{fix}}$$

In this case, the expression is identical to the NC-average model, except for the correction factor $(1-f_0)$. Therefore, the result depends strongly on the single-mutant fixation probability $P_{p_1}^{\text{fix}}$ and thus the selective advantage of p_1. Note that in this limit, our predicted fixation time depends very sensitively on f_0, which approximates the polymorphic part of the population and is not exact - the quantitative impact of this will be investigated in detail in section S5.2.

2. If $t_c \gg t_p$, most fixations take place when the prevalent genotype g_0 is a portal genotype to p_1. In this case, the bursty and overdispersed aspect of variation dominates the outcomes and we have

$$\begin{aligned} t_1^{\text{fix}} &\approx t_p \\ &= t_{ne}/(\phi_{p_1 p_0} L(K-1) P_{\text{portal } p_1}^{\text{fix}}) \end{aligned}$$

In this case, the time only depends on the selective advantage via $P_{\text{portal } p_1}^{\text{fix}}$, which is only sub-linearly affected by changes in the selective advantage if bursts are large (see Eq. S14 and Fig S1). In agreement with the arguments made by McCandlish [15], a higher population size does not accelerate fixation in this case (since t_{ne} is independent of population size and $P_{\text{portal } p_1}^{\text{fix}}$ can saturate). The underlying reason is that the rate-limiting step is the random drift onto a portal genotype, which is independent of population size N and selective advantage s_1.

S1.3.5 Fixation probability of two competing mutants (Fig 7 in the main text)

As before, in the NC-average model, we first estimate the fixation times for both mutants and then calculate the probability that p_r fixes first as $P_{\text{r fixes}} = (1 + t_r^{\text{fix}}/t_f^{\text{fix}})^{-1}$. The only difference is that the random-map calculations (Eq. S15) are used to compute these fixation times, rather than relying on the average-rate calculations (Eq. S9).

S2 Additional data on overdispersed variation in RNA

S2.1 Existence of bursts for a range of target phenotypes

In the main text, we only plotted the overdispersed statistics for one phenotypic transition in Fig. 4. Here, we show this data for further phenotypic transitions from the same initial NC (Fig S2). We restrict ourselves to phenotypes that appear at intermediate rates ($0.01 \leq \phi_{p_i p_0} \leq 0.05$, excluding the unfolded structure) since these appear at sufficiently high rates to collect sufficient data for a bar chart, but at a sufficiently low rate that they are not produced continuously. We find that the appearance of new phenotypes p_i is also overdispersed for these further choices of p_i.

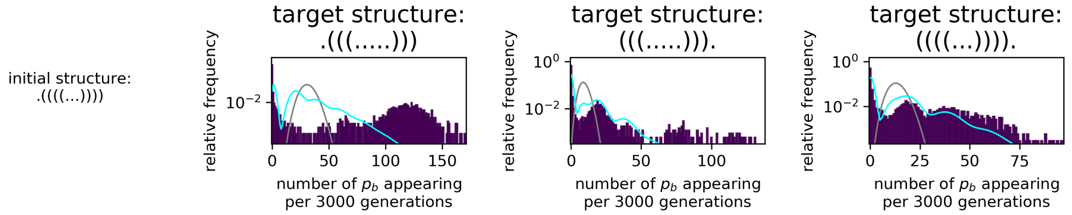

Figure S2: ***Strong deviations from Poisson statistics for the appearance of phenotype*** $\mathbf{p_b}$ ***in a population neutrally evolving with stabilising selection for*** $\mathbf{p_g}$. *As in Fig 4 in the main text, the appearance of novel phenotypes is quantified by splitting the simulation into time intervals of* $\Delta t = 3000$ *generations and recording how often a given new phenotype* p_b *appears in each* Δt. *This data is for the RNA GP map only. The number of appearances per interval is highly overdispersed compared to a Poissonian distribution with the same mean (grey line), which would be observed in the average-rate model. The overdispersion is also higher than analytically predicted for the random map (cyan line), which is not surprising given that the RNA GP map includes non-neutral correlations that do not exist in the random map. Parameters: population size* $N = 1000$, *mutation rate* $u = 2 \times 10^{-5}$, *total time* 10^7 *generations. The initial NC is the one shown in Fig 1 in the main text, and the target phenotypes* p_b *are given above each plot in dot-bracket notation (the first of the three plots has the same target as Fig 4 in the main text).*

S2.2 Dependence of burstiness on population parameters - more detailed plots

In the main text, we considered simulations for a range of population parameters (i.e. changing mutation rates u and population sizes N) and summarised the evolutionary simulation by a single number, the coefficient of variation in the time intervals between two p_i appearances. We found coefficients of variation greater than one, indicating overdispersion. The underlying datasets are visualised in more detail in Figs. S3 & S4: for each combination of GP map model and population parameters, we show the data as a histogram (similar to Fig 4 in the main text and Fig S2 above). We find that the distributions are indeed overdispersed, with overdispersion decreasing in the highly polymorphic or high-drift limit. As before, the overdispersion becomes clearer as we go from the random null model to the more inhomogeneous RNA map. Moreover, we find, that for the random GP map, the distributions are well-approximated by our analytic calculations, except for highly polymorphic populations with $NuL > 1$, where our underlying assumptions about a well-localised population with a single prevalent genotype and long t_{ne} are expected to break down.

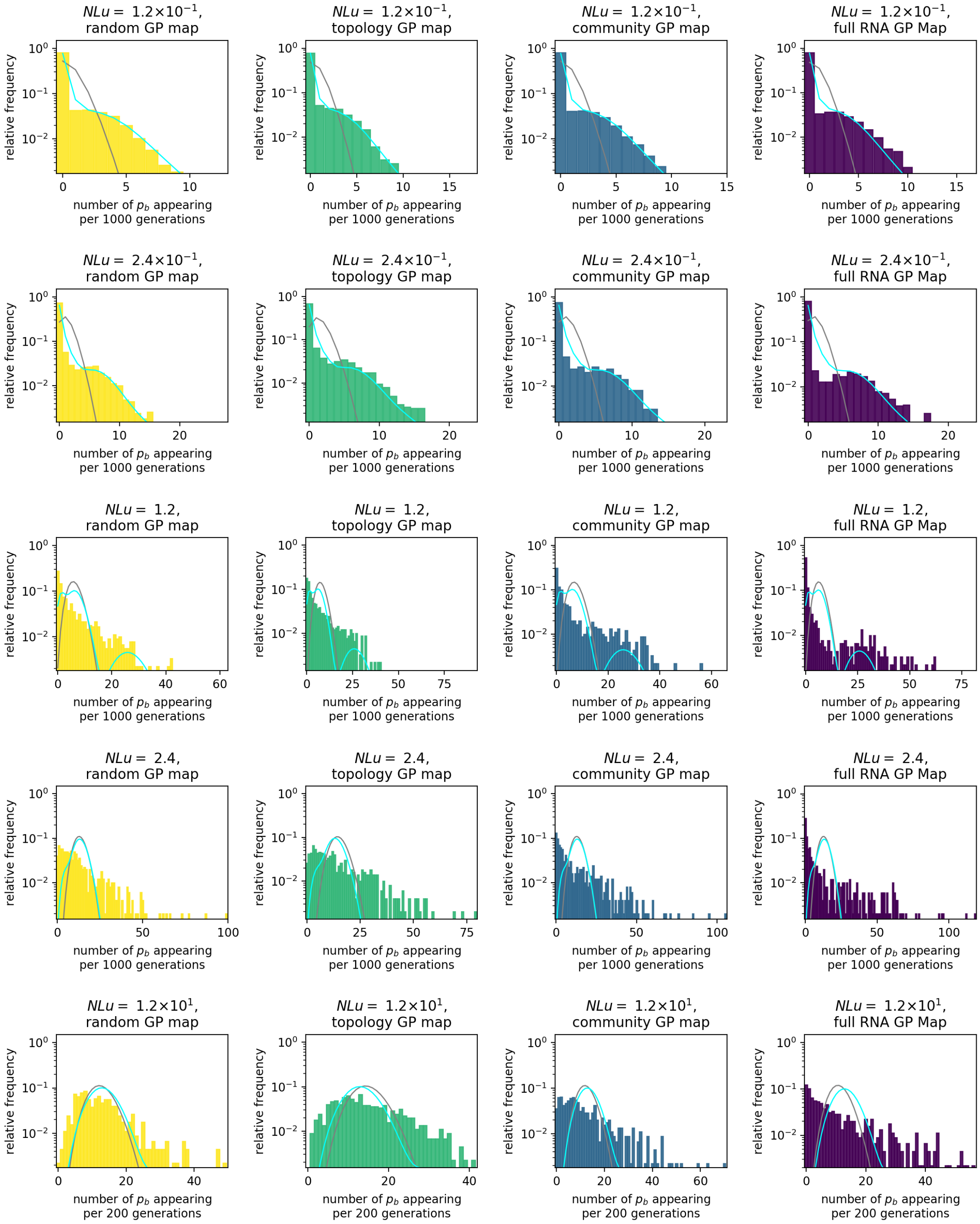

Figure S3: ***Histogram representation of the data underlying Fig 5A in the main text:*** *the simulation data for each combination of the parameter 'mutation supply' NuL (row) and 'type of GP map' (column) is visualised as a histogram, as before in Fig 4 in the main text and Fig S2 above. As expected, the analytic approximation breaks down for highly polymorphic populations with $NuL > 1$ since the underlying assumptions are no longer valid in this case. Note that this way of visualising the data requires us to pick Δt, but a 'good' choice of Δt depends both on the neutral fixation time and the total simulation time, and thus on the parameter 'mutation supply' NuL. Here, we nevertheless used a constant $\Delta t = 1000$ for comparability, except for the last row, where the total simulation time was shortest and had to be split into shorter interval to collect enough statistics for the histograms.*

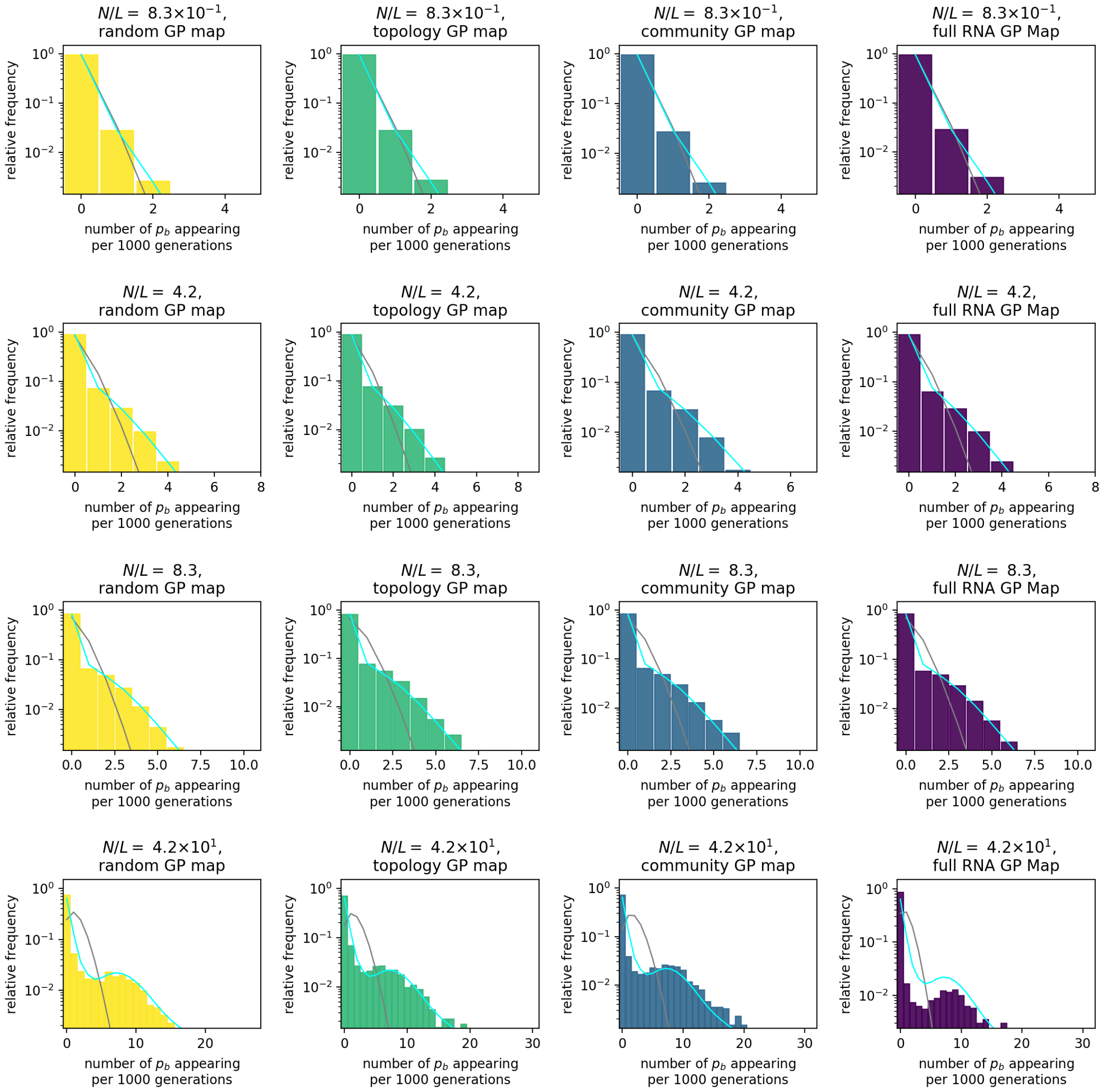

Figure S4: ***Histogram representation of the data underlying Fig 5B in the main text:*** *the simulation data for each combination of the parameter 'population size/sequence length' N/L (row) and 'type of GP map' (column) is visualised as a histogram, as before in Fig 4 in the main text and Fig S2 above.*

S2.3 Dependence of burstiness on population parameters for a range of initial conditions

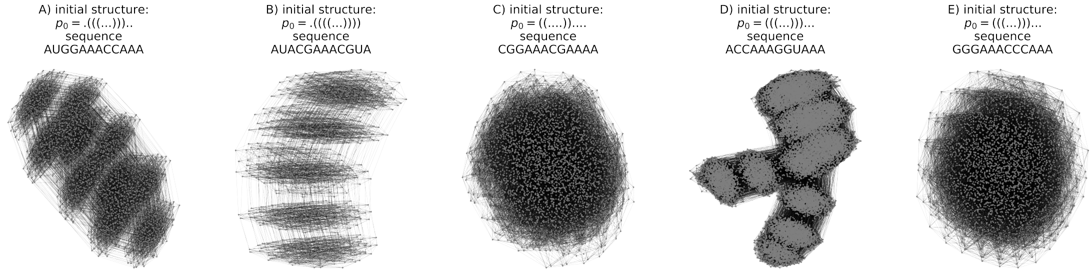

Figure S5: ***The NCs used as starting points in the simulations of evolving populations:*** *these five NCs were randomly chosen from all large NCs (with $> 10^3$ genotypes). Each NC is one connected network within the neutral set of a given phenotype and is uniquely defined once we know one sequence in the NC (then any other sequence that can be reached through a series of neutral mutations from that sequence is part of the NC). Thus, the title of each network includes both the RNA structure associated with the neutral set in dot-bracket notation and one sequence to identify the NC. The NC in Figs 1 &3-6 of the main text is the second one. The graphs are drawn using the force-layout algorithm from NetworkX [8].*

In the main text, we only had space to present the coefficient of variation (as a proxy for burstiness) for a single initial NC and a single target phenotype. Here, we repeat the same analysis for populations starting at all five NCs from Fig S5. These five initial NCs, which include the NC from the main text, are randomly chosen from all large NCs (with $> 10^3$ genotypes). We only consider large NCs for the following reasons:

- While small and large NCs can have phenotypic transitions with high ϕ_{pq}, only large NCs have phenotypic transitions with small ϕ_{pq} since low-frequency transitions can only exist if they occur a small number of times in a large NC. Thus, large NCs are needed to explore phenotypic transitions with a range of ϕ_{pq} values.
- The randomisation employed in creating the different models essentially shuffles the mutational neighbourhood of an NC and this is more effective in large NCs.
- Because phenotypes with large neutral sets are more likely to appear in nature [2, 4, 5], large neutral sets and therefore large NCs are likely to play an important role in evolution.
- Neutral set sizes and NCs for biologically realistic sequence lengths of $L > 12nts$ are likely to be even larger, so it is important to understand the dynamics on large NCs.

For each of these initial NCs, we studied the overdispersion for a variety of target phenotypes: this data is shown in Figs S6 - S10, one figure for each initial NC. For each of the five starting NCs, the set of target phenotypes was chosen according to the following criteria: we need phenotypes that are frequent enough that they appear sufficiently often in the simulation to estimate their statistics. However, we also need them to be rare enough that they are unlikely to appear in every generation since this could lead to confounding effects: we would have to take into account the discrete nature of generations and we would need a sophisticated treatment for concurrent appearances in the same generation, which we currently simply count as a single event. Therefore, data was plotted for all target phenotypes with an intermediate frequency ($0.01 \leq \phi_{p_i p_0} \leq 0.05$). Out of this set, we applied additional criteria before including a given target phenotype in the plot: we checked that the phenotype appears at least 500 times in every simulation, and at most every five generations (using the median time interval to apply this criterion), to ensure sufficient data and to avoid problems with discrete generation times.

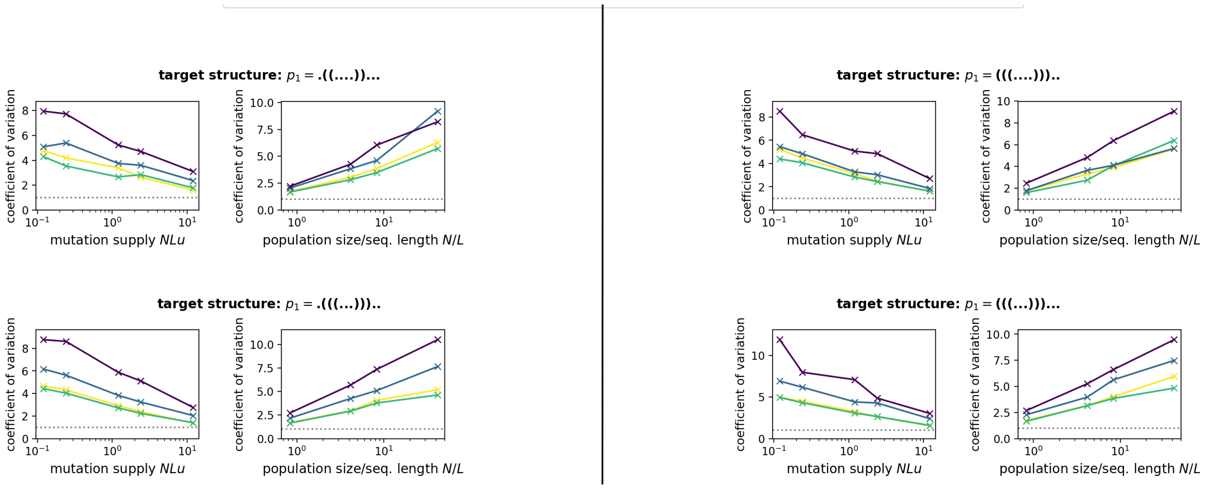

Figure S6: ***How does the amount of overdispersion, quantified by the coefficient of variation, depend on population parameters?*** *This figure shows the same analysis as Fig 5 in the main text: as a measure of burstiness, the coefficient of variation of the time between two p_1 appearances is shown. Here, the initial NC and phenotype is the one in Fig S5B (the one used in the main text). The target phenotype p_1 is a different one in each row and indicated in the row title. For each target phenotype, there are two analyses: (left) a range of mutation rates u (and thus NLu) for a fixed population size of $N = 200$ and (right) a range of population sizes N (and thus N/L) for a fixed mutation rate of $u = 5 \times 10^{-5}$.*

The results in Figs S6 - S9 are in agreement with those shown in the main text: the overdispersion tends to be weakest on the random map and strongest on the RNA GP map due to its highly inhomogeneous structure. Furthermore, the overdispersion tends to decrease with increasing genetic drift (small population size N) or increasing genetic diversity (large uLN), consistent with the scaling arguments in the main text and ref [17]. Note that it is difficult to capture a complex phenomenon like overdispersion in a simple parameter: there are limitations in quantifying burstiness of finite data sets using simply metrics based on the coefficient of variation (see for example [10]); and thus, small quantitative differences in the coefficient of variation may not be significant, and reliable conclusions can only be reached by also investigating the full distributions, as we did in Fig 4 in the main text and Fig S2.1 in this document.

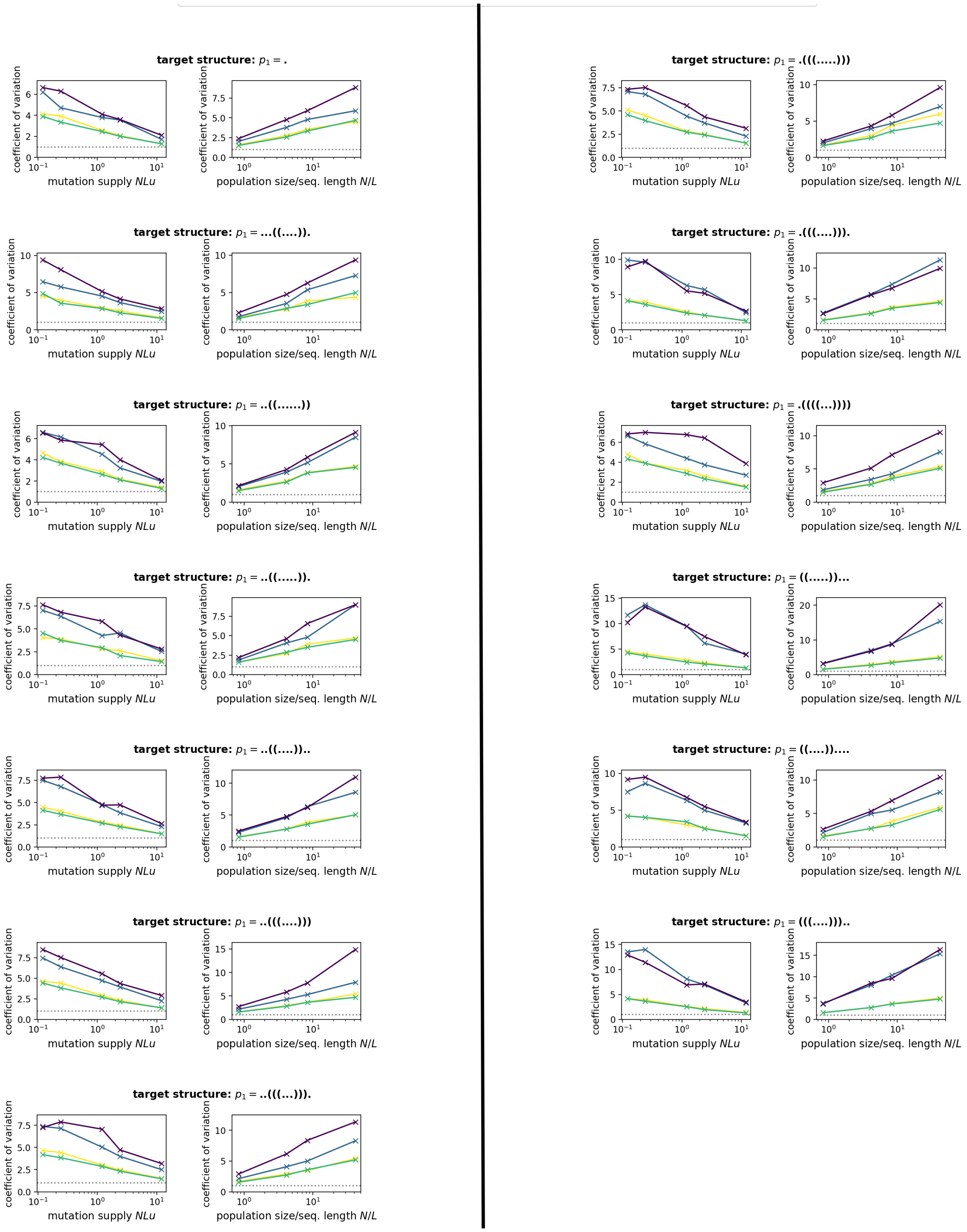

Figure S7: Same as Fig S6, but here the initial NC and phenotype is the one in Fig S5A.

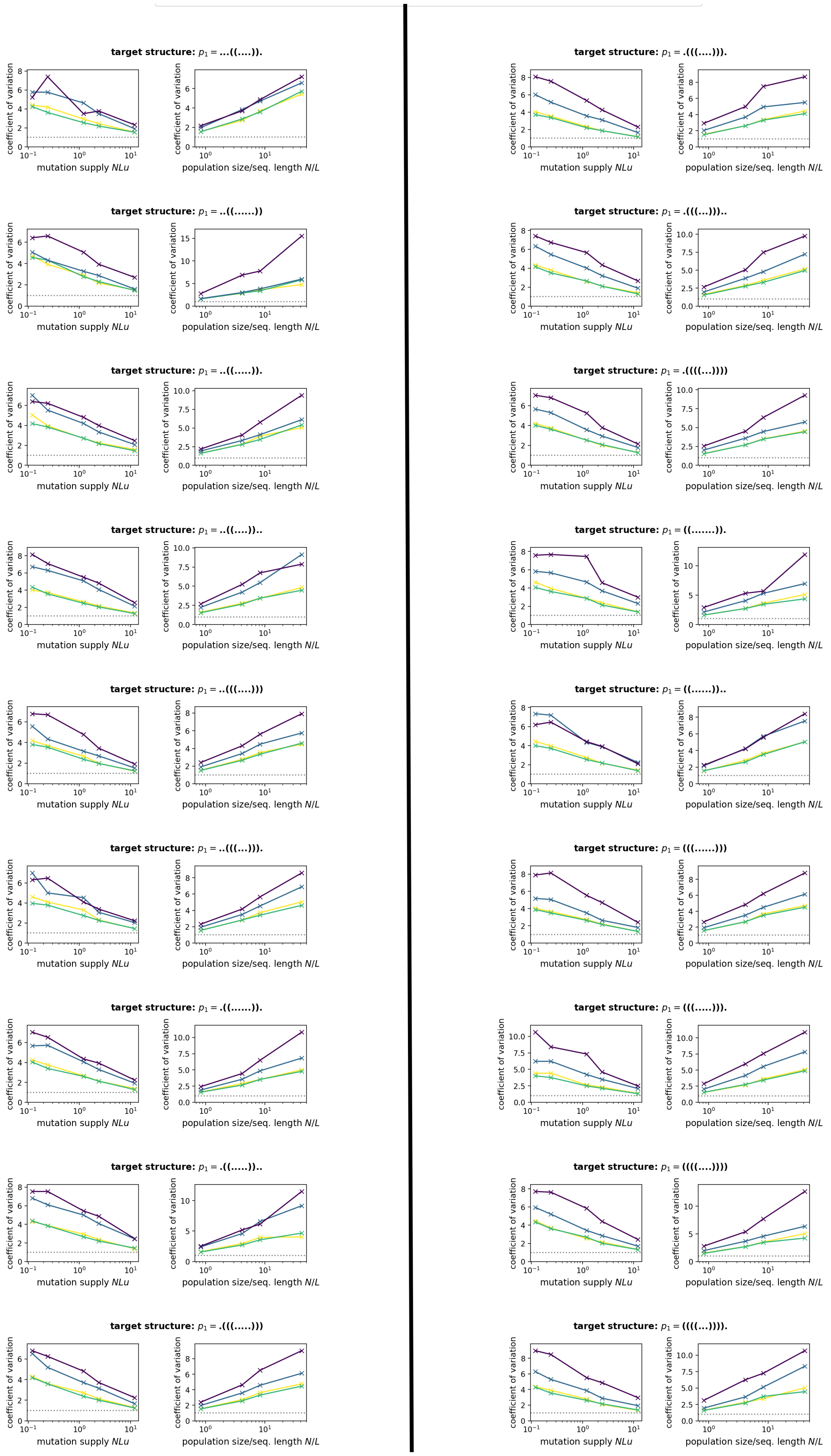

Figure S8: Same as Fig S6, but here the initial NC and phenotype is the one in Fig S5C.

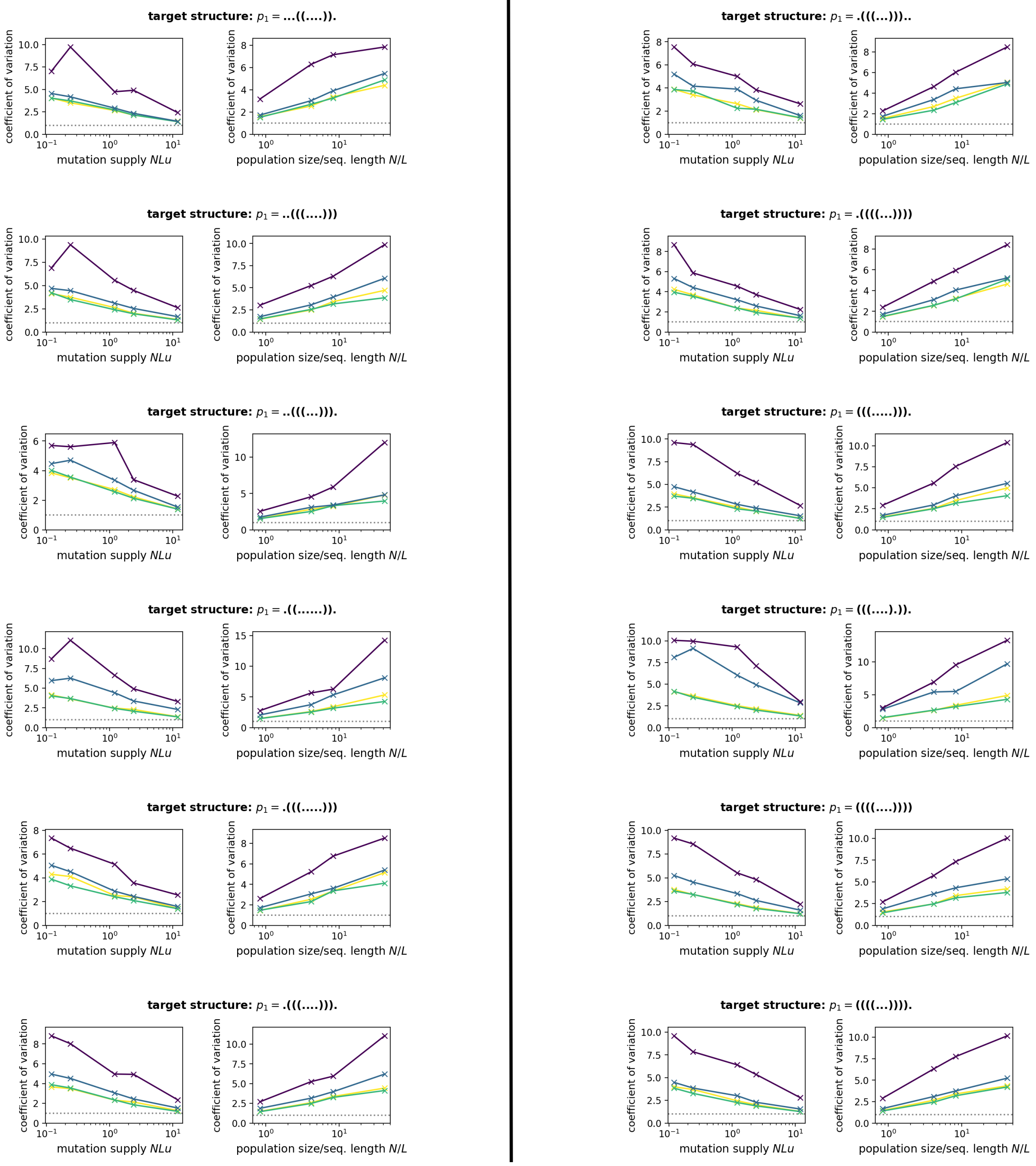

Figure S9: Same as Fig S6, but here the initial NC and phenotype is the one in Fig S5D.

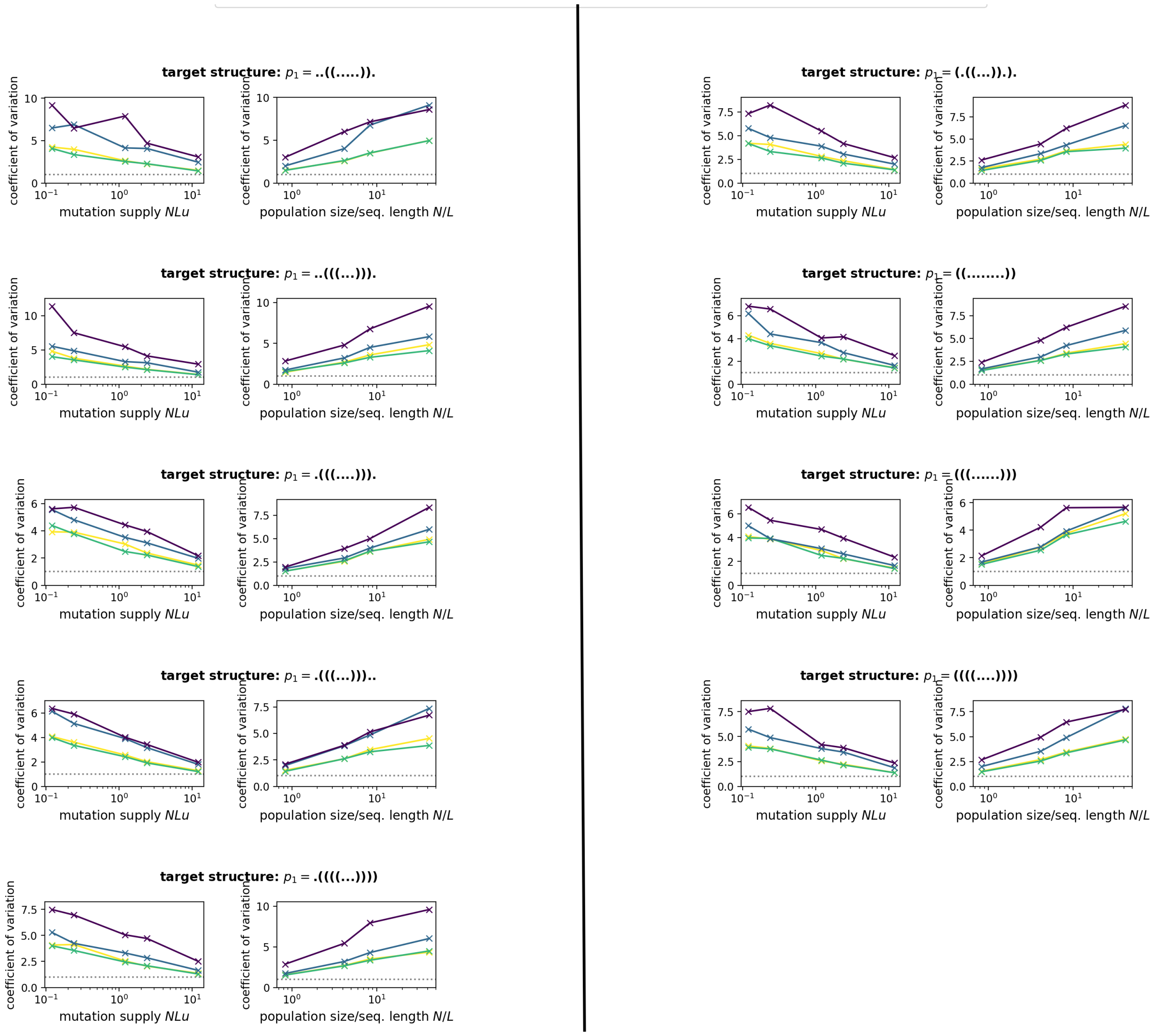

Figure S10: Same as Fig S6, but here the initial NC and phenotype is the one in Fig S5E.

S2.4 Longer sequence length of L = 30 nt

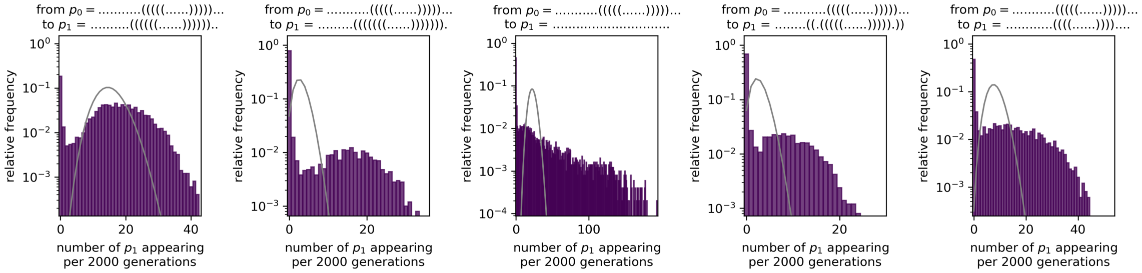

Figure S11: ***Overdispersion for RNA structures of length*** $L = 30$ ***nt:*** *as in Fig 4 in the main text, we simulated a population under strong stabilising selection, and counted how many times a new phenotype* p_1 *is introduced in a time interval of* $\Delta t = 2000$ *generation. We find that* p_1 *appears in a bursty manner compared to a Poissonian distribution with the same mean (grey line), which would be expected in a constant-rate process. Parameters: population size* $N = 1000$*, mutation rate* $u = 1 \times 10^{-5}$*, total time* 10^7 *generations. In each subplot, the initial and final structure are given in the plot titles - the initial structure* p_0 *is the same throughout this plot since all data is derived from a single evolutionary simulation, where selection stabilises this specific structure. For final structure* p_1*, we restricted ourselves to structures that are expected to appear with intermediate frequencies of* $0.001 \leq \phi_{p_1 p_0} \leq 0.02$*, since our simulations are not long enough to collect sufficient data for low-frequency structures (we only plot data for structures that appear at least* 10^3 *times and on average more than twice in each* Δt*). To apply this criterion, we approximate the* $\phi_{p_1 p_0}$ *values using the procedure from our previous work [13], which uses the site-scanning sampling method [19].*

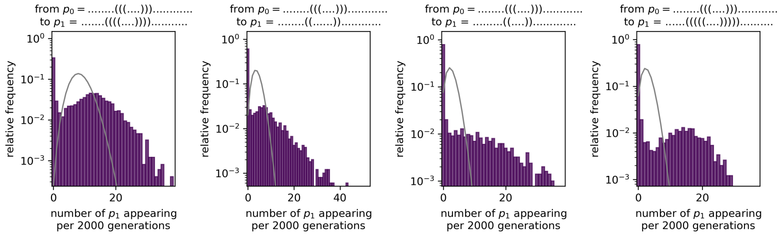

Figure S12: ***Overdispersion for RNA structures of length*** $L = 30$ ***nt:*** *same as Fig S11, but for a different initial structure* p_0*.*

Throughout the main text, we worked with RNA sequences of length $L = 12$ nt and their secondary structures. This allowed us to visualise entire neutral spaces and create null models with the same statistics. Such a full analysis is not feasible for much longer sequence lengths, since the number of sequences that would have to be folded to gain a full overview of the GP map grows exponentially as 4^L. However, we expect bursts to play a role for longer sequence lengths as well: sophisticated sampling methods have shown that NCs still have a pronounced community structure for sequences up to length $L = 45$ nt [18], indicating that NCs remain inhomogeneous, and non-neutral correlations have been shown for sequences up to length $L = 20$ nt [7]. Even without this inhomogeneous structure, i.e. for the random map, we expect overdispersion under the conditions outlined in the main text.

Here, we simulate evolutionary processes for sequences of length $L = 30$ nt, which is too long for an exhaustive analysis since the number of sequences of that length is $4^{30} \approx 10^{18}$. Figs S11 - S15 show that the key result from the main text remains unchanged: new phenotypes p_i appear in overdispersed bursts. These figures show data for initial structures with between one and four stacks - since the number of stacks in a structure is closely linked to its neutral set size [4] (and

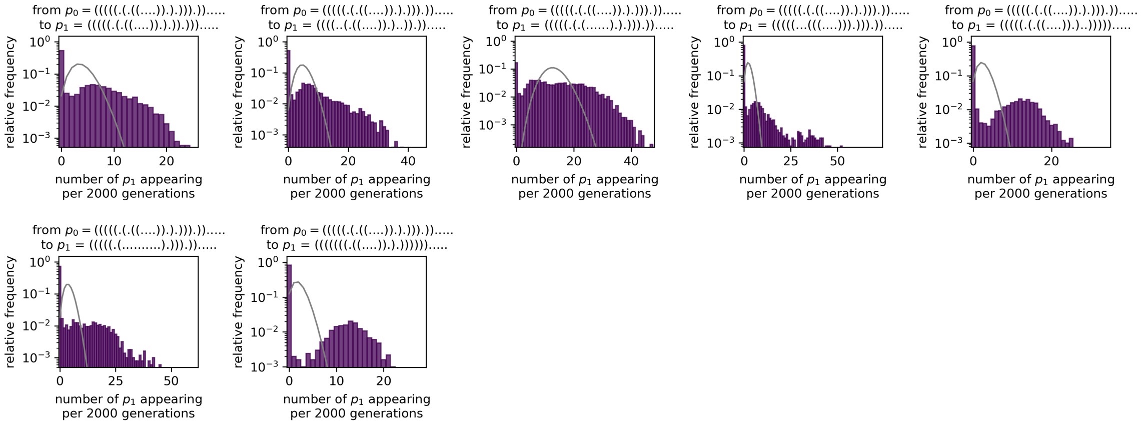

Figure S13: ***Overdispersion for RNA structures of length*** $L = 30$ ***nt:*** *same as Fig S11, but for a different initial structure* p_0.

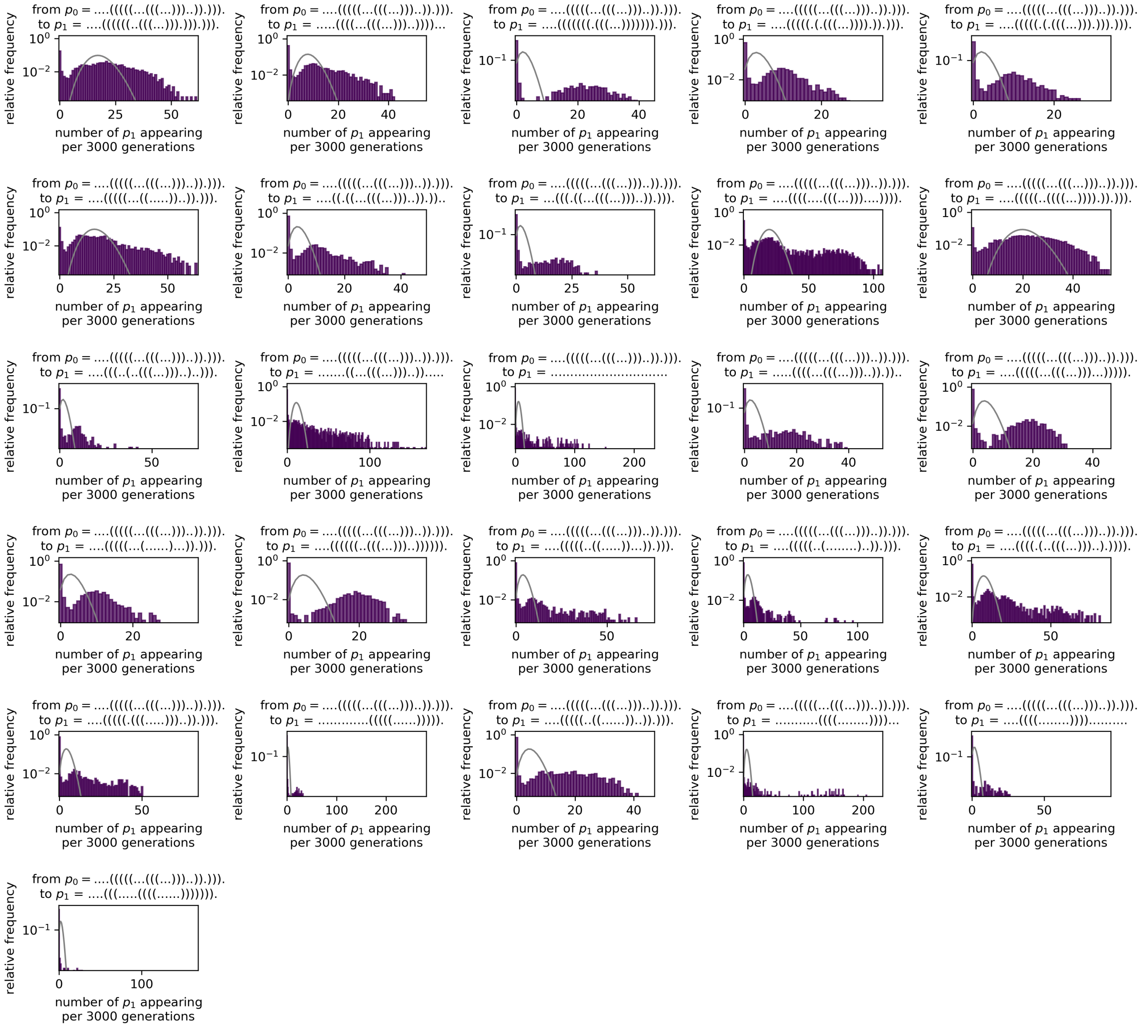

Figure S14: ***Overdispersion for RNA structures of length*** $L = 30$ ***nt:*** *same as Fig S11, but for a different initial structure* p_0.

thus mutational robustness [7] and folding stability [12]), including a range of number of stacks ensures a diverse set of structures (as in our previous work [13]).

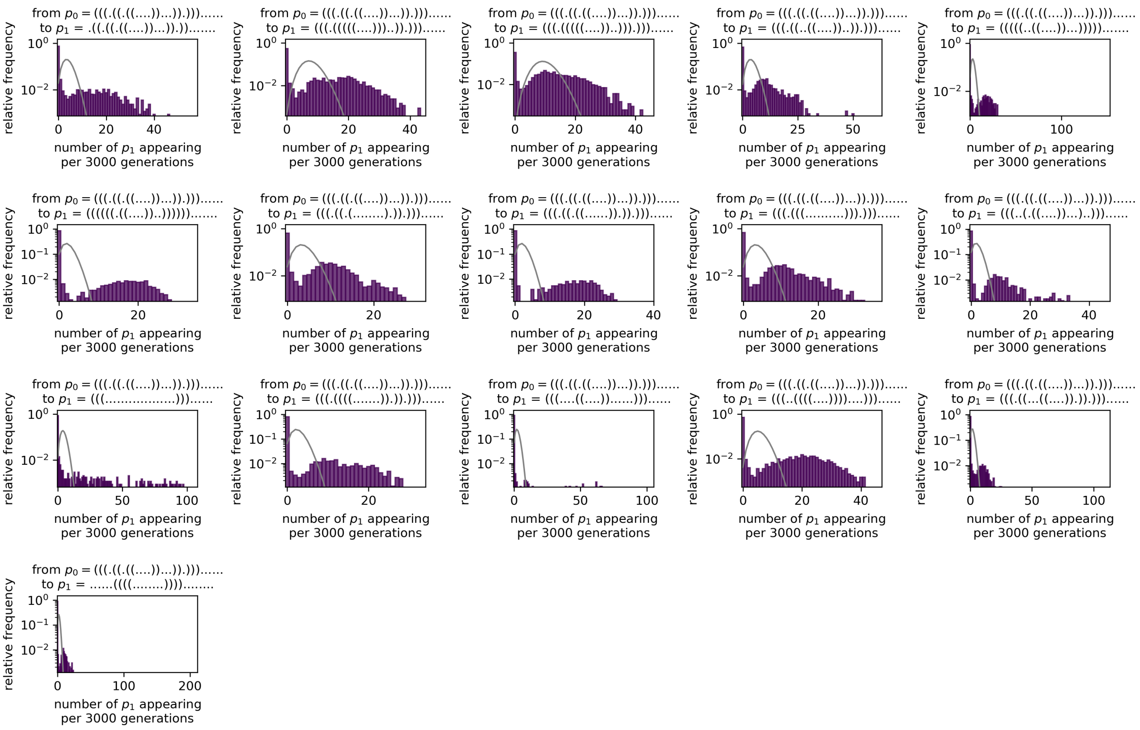

Figure S15: ***Overdispersion for RNA structures of length*** $L = 30$ ***nt:*** *same as Fig S11, but for a different initial structure* p_0*.*

S2.5 Theoretical predictions of fixation times for sequence lengths L=50 nt and L=500 nt

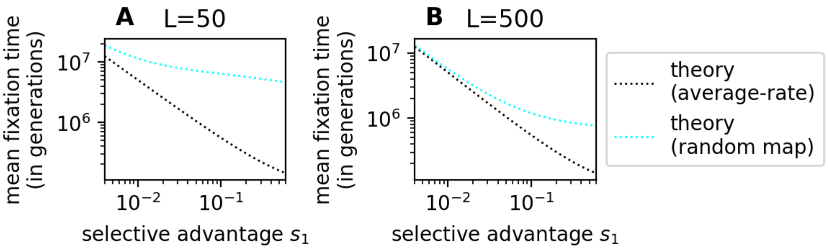

Figure S16: ***Fixation time calculations for different sequence lengths*** L***:*** *these plots show analytic predictions for RNA sequences of length (A)* $L = 50$ *nt and (B)* $L = 500$ *nt, modelling the same scenario as Fig. 6 in the main text. We predict analytically, how many generations it takes until* p_1 *fixes for two cases: first, when* p_1 *appears with a constant probability per generation (grey line, given by Eq. S9), and secondly, we repeat the calculation for the corresponding random GP map (teal line, given by Eq. S15), where there are several discrete genotypes in each neutral space and each genotype's mutational neighbourhood is drawn from a fixed distribution. In all cases,* p_1 *fixes more rapidly if its selective advantage is higher, but this decrease is steeper for the constant-rate model than in the random map calculation, which has overdispersed dynamics. Parameters: population size* $N = 10^3$*, mutation rate* $u = 10^{-4}/L$ *(to keep the mutation supply constant across the figure), average rate of mutating to* p_1 $\phi_{p_1 p_0} = 1 \times 10^{-4}$*, robustness of the initial phenotype* $\rho_{p_0} = 0.1$*. These parameters lead to burst sizes of* $M = N/((K-1)\rho L)$ *of 66.7 for* $L = 50$*, and* $N = 6.67$ *for* $L = 500$*. As expected, clear deviations from the average-rate model emerge for selection coefficients larger than about* $1/M$*. The lowest selective advantage considered is* $4/N$ *since we are not interested in effectively neutral mutations with* $s \lessapprox 1/N$*.*

To further investigate, whether our results generalise to longer sequences, we apply our analytic approximations for fixation times in the random GP map to sequences of length $L = 50$ nt and $L = 500$ nt (Fig S16). As before, we find that fixation times can differ by many orders of magnitude between the average-rate prediction and the prediction for the random GP map.

S3 Overdispersed variation in other GP maps

S3.1 Overdispersed variation in a lattice model for protein tertiary structure

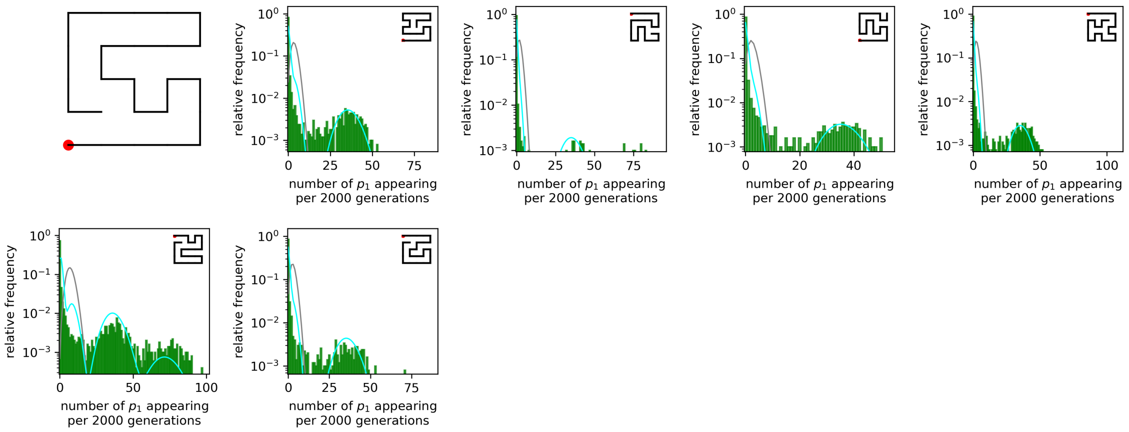

Figure S17: ***Overdispersion on a lattice protein model GP map:*** *as in Fig 4 in the main text, we simulated a population under strong stabilising selection, and counted how many times a new phenotype* p_1 *is introduced in a time interval of* $\Delta t = 2000$ *generation. Our results show that* p_1 *introductions are highly overdispersed compared to a Poissonian distribution with the same mean (grey line), which would be observed in a constant-rate process. The analytic prediction for the random map is shown in cyan. Parameters: population size* $N = 1000$*, mutation rate* $u = 2 \times 10^{-5}$*, total time* 10^7 *generations. The initial structure* p_0 *(shown in the first subplot) is the same throughout this plot since all data is derived from a single evolutionary simulation with stabilising selection for this initial structure. The target structure for each subplot is shown in the top-right corner. We restricted ourselves to target structures that are expected to appear with intermediate frequencies (*$0.001 \leq \phi_{p_1 p_0} \leq 0.05$*) since our simulations are not long enough to collect sufficient data for low-frequency structures (we only plot data for structures that appear at least* 500 *times and on average at least twice in each* Δt*).*

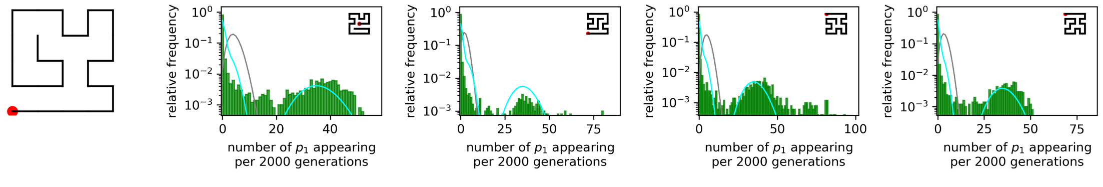

Figure S18: ***Overdispersion on a lattice protein model GP map:*** *same as Fig S17, but for a different initial structure (shown in the first subplot).*

Because proteins and their folded structures play a central role in molecular biology, we also test our hypothesis on a simple model of protein tertiary structure, the well-studied [1, 7, 11] HP ('hydrophobic-polar') lattice model. This model simulates molecular chains of hydrophobic and polar residues on a lattice. The chains fold into the structure that maximises the number of energetically favourable contacts, in this case between hydrophobic residues, and thus simulate one important aspect of protein folding. Here, we use the Python implementation and data for

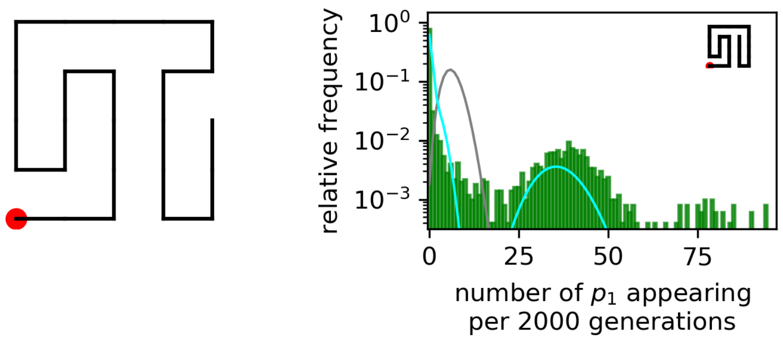

Figure S19: ***Overdispersion on a lattice protein model GP map:*** *same as Fig S17, but for a different initial structure (shown in the first subplot).*

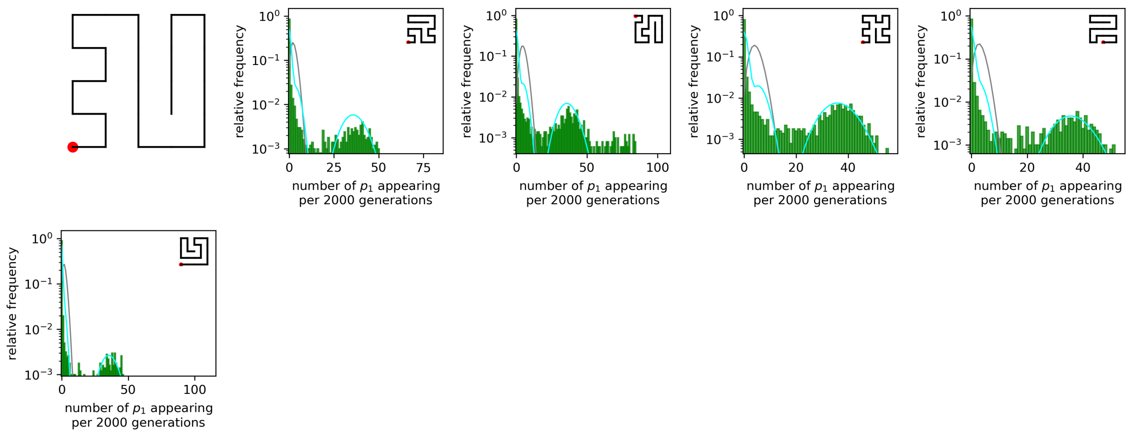

Figure S20: ***Overdispersion on a lattice protein model GP map:*** *same as Fig S17, but for a different initial structure (shown in the first subplot).*

compact 5×5 HP lattice proteins from our previous work [13].
As before for the RNA map, we find that phenotypic variation in the HP protein model is overdispersed (see Figs S17 - S20). However, unlike for RNA, this overdispersion is not much higher than that predicted by our analytic approximation for a random map without genetic correlations. This is consistent with previous observations that non-neutral correlations, which would amplify the bursts compared to the random map, are weaker in the HP protein model than in the RNA GP map [7]. Whether this is an artefact of the coarse-grained nature of the HP model and its energetics, or whether non-neutral correlations are truly weaker in proteins than in RNA, is a question for future research.

S3.2 Overdispersed variation in Richard Dawkins's biomorphs, a toy model of development

As a second test, we work with a GP map that is based on completely different principles than the molecular RNA and HP models explored so far: Richard Dawkins's biomorphs. Biomorphs are a simple toy model of growth processes and are based on recursive branching rules, which mimic developmental processes in nature [3]. Biomorphs have a genotype, which is made up of nine integer values, and from this genotype, a 2D drawing is produced as a phenotype. This 2D drawing can be converted into a discrete, clearly defined phenotype, using methods in ref [14]. Here, we simulate an evolving population under stabilising selection on the biomorphs GP map to investigate, whether we observe bursts in the introduction of new phenotypes. Figs S21-S26 shows that we do indeed find such bursts. Unfortunately, as explained in more detail in the main

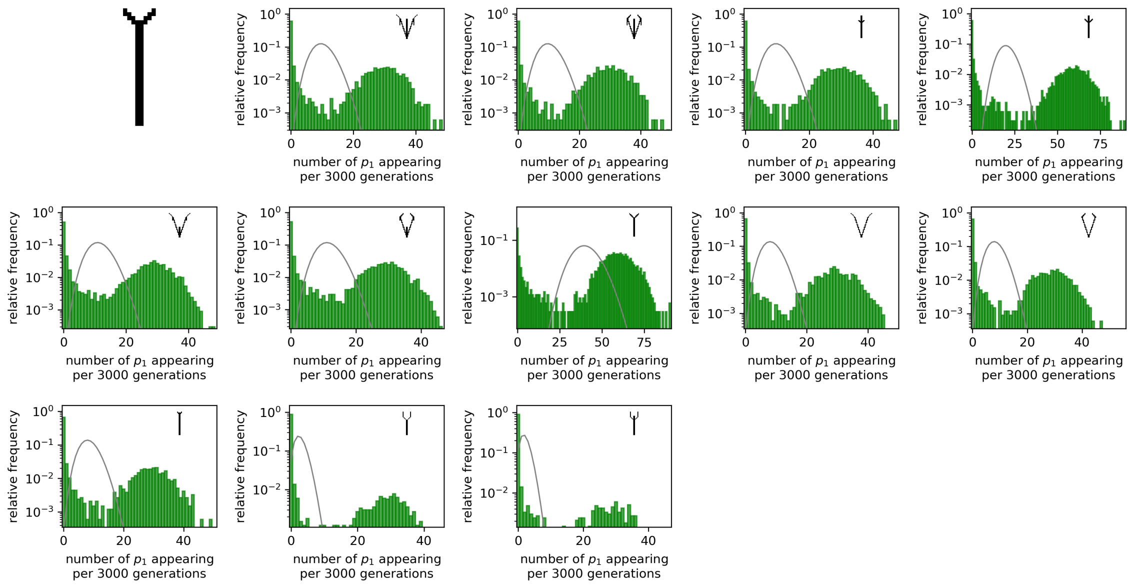

Figure S21: ***Overdispersion on the GP map of Richard Dawkins's [3] biomorphs:*** *as in Fig 4 in the main text, we simulated a population under strong stabilising selection and counted how many times a new phenotype* p_1 *is introduced in a time interval of* $\Delta t = 3000$ *generation. Our results show that* p_1 *introductions are highly overdispersed compared to a Poissonian distribution with the same mean (grey line), which would be observed in a constant-rate process. Parameters: population size* $N = 1000$, *mutation rate* $u = 2 \times 10^{-5}$, *total time* 10^7 *generations. The initial phenotype* p_0 *(shown in the first subplot) is the same throughout this plot since all data is derived from a single evolutionary simulation, where this initial phenotype is under stabilising selection. The target phenotype for each subplot is shown in the top-right corner. We restricted ourselves to target structures that are expected to appear with intermediate frequencies* ($0.001 \leq \phi_{p_1 p_0} \leq 0.05$) *since our simulations are not long enough to collect sufficient data for low-frequency structures (we only plot data for structures that appear at least* 500 *times and on average at least twice in each* Δt*).*

text, we cannot apply our analytic approximation for the random map since the biomorphs GP map has a somewhat unusual genotype space connectivity.

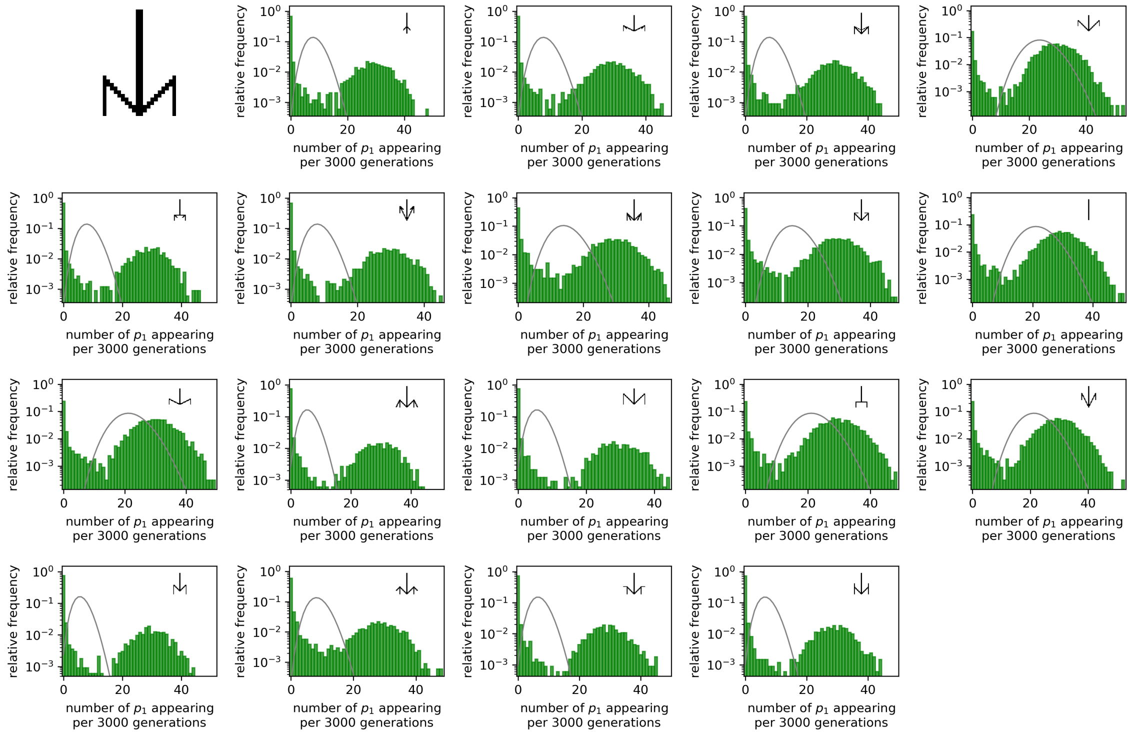

Figure S22: ***Overdispersion on the GP map of Richard Dawkins's [3] biomorphs:*** *same as Fig. S21, but for a different initial phenotype.*

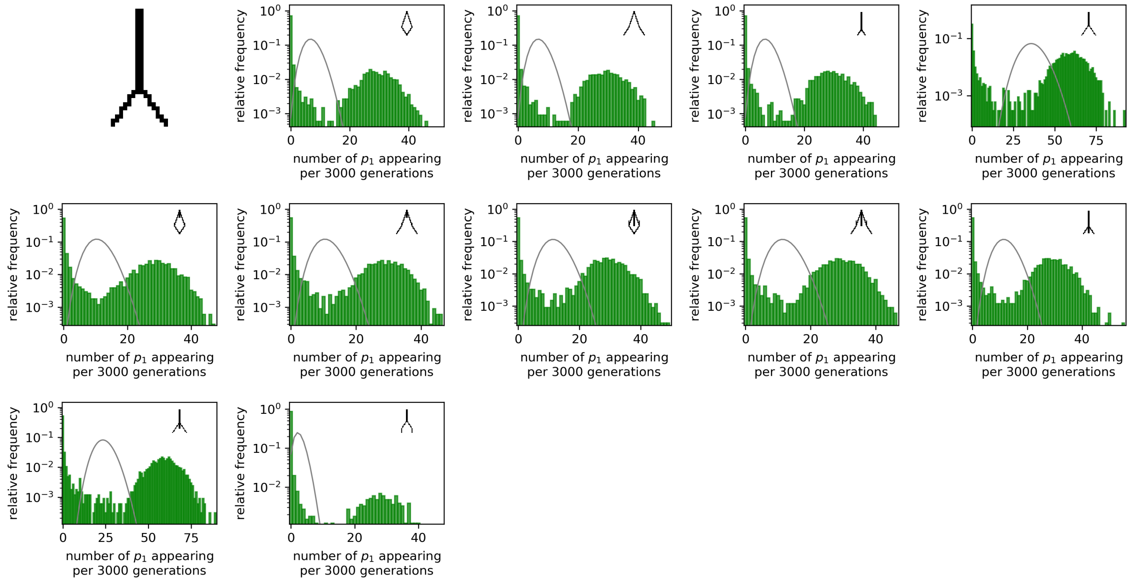

Figure S23: ***Overdispersion on the GP map of Richard Dawkins's [3] biomorphs:*** *same as Fig. S21, but for a different initial phenotype.*

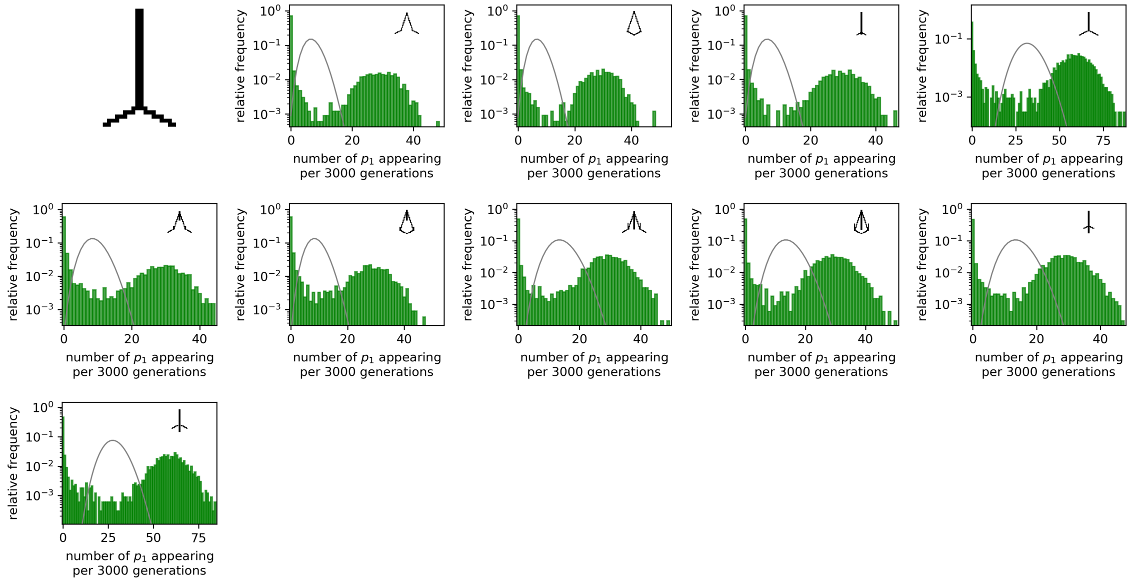

Figure S24: ***Overdispersion on the GP map of Richard Dawkins's [3] biomorphs:*** *same as Fig. S21, but for a different initial phenotype.*

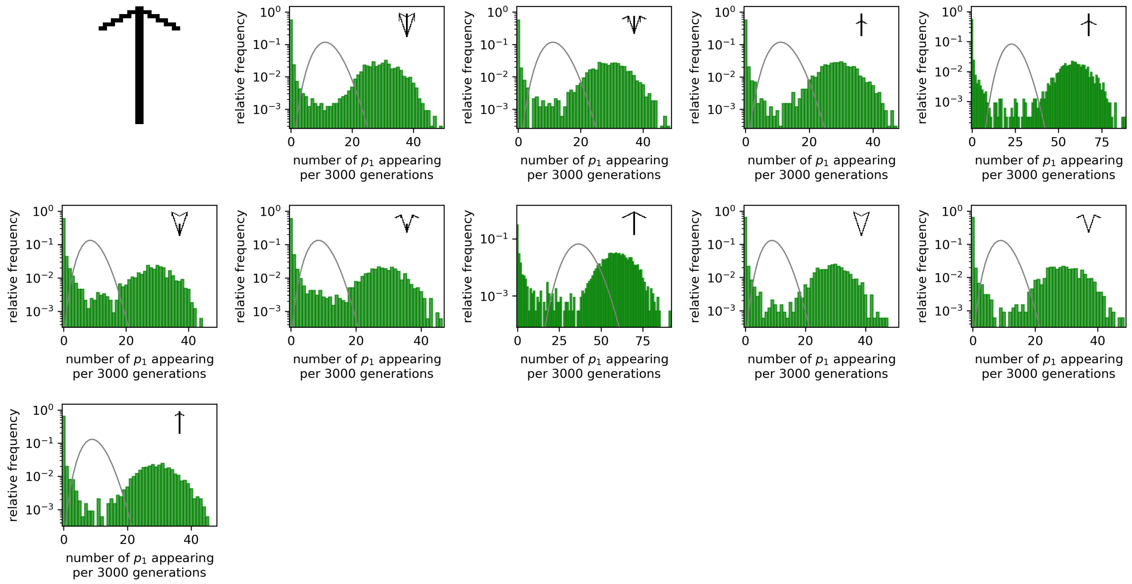

Figure S25: ***Overdispersion on the GP map of Richard Dawkins's [3] biomorphs:*** *same as Fig. S21, but for a different initial phenotype.*

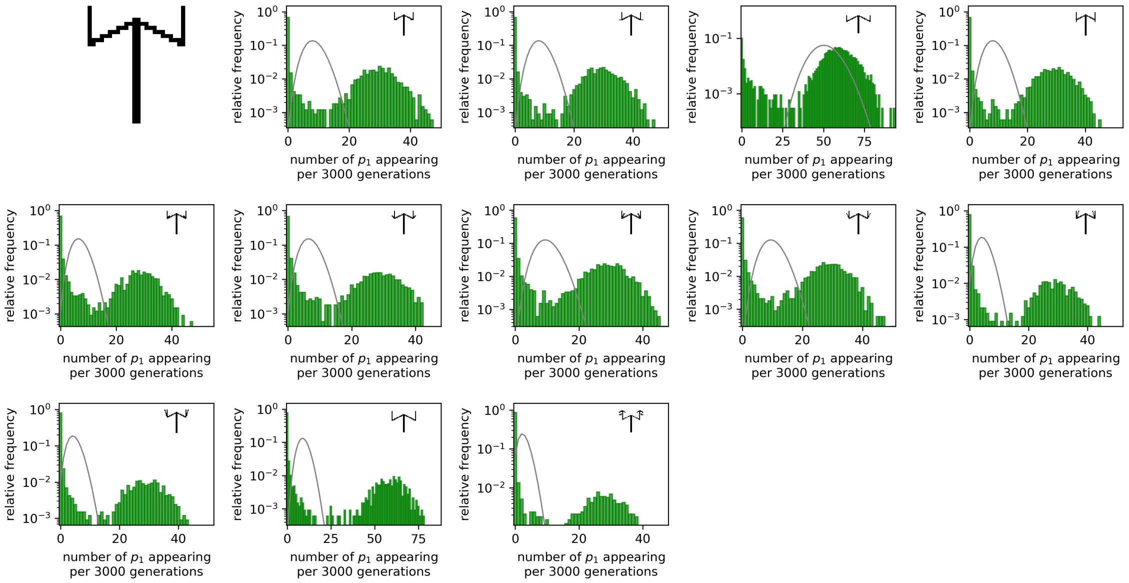

Figure S26: ***Overdispersion on the GP map of Richard Dawkins's [3] biomorphs:*** *same as Fig. S21, but for a different initial phenotype.*

S4 Effect of quasi-simultaneous p_1 mutants during a burst

In all of our calculations and arguments in this paper, we have used the single-mutant fixation probability for each new phenotypic introduction. However, in the bursty case, p_1 mutants can arise at high rates during a burst, which could increase the likelihood that two p_1 mutants arise in quick succession or even in the same generation. To understand if this has any effect on fixation processes, we need to compare the probability of fixation for two limiting cases: (I) n phenotypes p_1 undergo independent fixation or extinction processes (i.e. one p_1 mutant either fixes or is lost before the next one appears) or (II) n phenotypes p_1 appear in a single generation.

S4.1 Analytic treatment using the diffusion approximation

First, let us investigate the difference between these two cases using the classic expression for the probability of fixation of n p_1 phenotypes with a selective advantage of s_1 in a population of N haploid organisms [6]:

$$P_{fix}(\text{n } p_1) = \frac{1 - e^{-2s_1 n}}{1 - e^{-2s_1 N}} \tag{S16}$$

Let us denote the denominator of Eq. S16 as:

$$a = \frac{1}{1 - e^{-2s_1 N}} \tag{S17}$$

This constant a is approximately ~ 1 in the limit of strong selection ($Ns_1 \gg 1$) and $\gg 1$ for weak selection ($Ns_1 \ll 1$).

Then we can write the probability of fixation for case (I), where n phenotypes p_1 undergo independent fixation or extinction processes, as:

$$P_{fix}(\text{n indep.} p_1) = 1 - (1 - P_1)^n \tag{S18}$$

$$= n\ P_1 - \binom{n}{2}\ P_1^2 + O(P_1^3) \tag{S19}$$

Here P_1 is the probability of fixation for a single mutant.

$$P_1 = a\ (1 - \exp(-2s_1)) \tag{S20}$$

On the other extreme, the probability of fixation for case (II), where n mutants of type p_1 appear in the population simultaneously, is:

$$P_{fix}(\text{n } p_1) = a\ (1 - \exp(-2ns_1)) \tag{S21}$$

$$= a(1 - (1 - \frac{P_1}{a})^n) \tag{S22}$$

$$= n\ P_1 - \binom{n}{2}\ \frac{P_1^2}{a} + O((\frac{P_1}{a})^3) \tag{S23}$$

In the limit of strong selection, we have $a \sim 1$, and so Equations S22 and S18 are identical up to the $O((\frac{P_1}{a})^2)$ terms. Therefore, the probability of fixation per mutant is the same, regardless of whether fixation processes are independent or simultaneous.

However, in the limit of weak selection, $a \gg 1$, Equations S18 and S22 are different. Their Taylor expansions, Equations S19 and S23, indicate that the fixation probability per mutant is higher when mutants arrive in groups. However, this difference is likely to be small since it scales with P_1^2, where P_1 is the probability of fixation for a single mutant, which is small in the limit of weak selection.

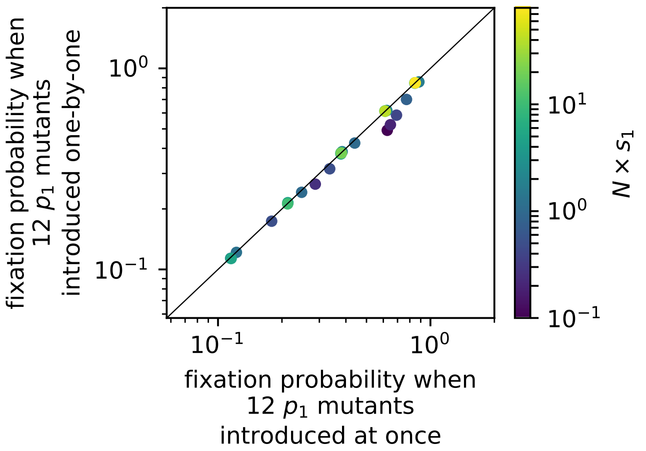

Figure S27: ***Does it make a difference whether p_1 mutants with a selective advantage of s_1 are introduced all at once in a single generation, or independently (i.e. after the last mutant has gone into fixation or to extinction):*** *we consider a range of population sizes N ($N = 20$, $N = 50$, $N = 100$, $N = 250$, $N = 500$, $N = 1000$) and selective advantages s_1 ($s_1 = 0.005$, $s_1 = 0.01$, $s_1 = 0.02$, $s_1 = 0.04$, $s_1 = 0.08$) and run two sets of simulations with a Wright-Fisher model: (1) 12 phenotypes p_1 undergo independent fixation or extinction processes (i.e. one p_1 mutant either fixes or is lost before the next one appears) or (2) 12 phenotypes appear in a single generation. We do not introduce any further mutations and simply run the simulation until the population only consists of p_0 (the initial phenotype) or p_1 (the new phenotype). We then estimate the probability of an all-p_1 outcome (i.e. a p_1 fixation) based on 10^5 such runs. We find, consistent with our analytic considerations (eqs S16-S23), that the fixation probability is approximately the same in both cases, except in the quasi-neutral limit $Ns_1 \lessapprox 1$, where p_1 is slightly more likely to fix if all p_1 mutants appear at once.*

S4.2 Simulations with concurrent or individual mutants

To test our analytic arguments, we simulate the fixation probabilities computationally for the two limiting cases: (I) n phenotypes p_1 undergo independent fixation or extinction processes (i.e. one p_1 mutant either fixes or is lost before the next one appears) or (II) n phenotypes appear in a single generation. We implement these scenarios as Wright-Fisher models without random mutations, such that all p_1 mutants are introduced in a controlled fashion, either all at once in the first generation or one by one (i.e. as soon as the last p_1 mutant has either been lost or fixed). We repeat this setup for a range of selective advantages s_1 and population sizes N and show the data in Fig S27. The results are consistent with our theoretical considerations: the fixation probability of p_1 does not depend strongly on whether all p_1 mutants are introduced at once in the first generation or one-by-one. Only in the very weak-selection, quasi-neutral mutation regime ($Ns_1 < 1$), can we observe a slight difference between the two scenarios: as predicted in our analytic calculations, in the quasi-neutral mutation regime, the fixation probability is higher if the mutants are introduced all at once. We can neglect this effect in the calculations in this paper since we are specifically interested in adaptation, not quasi-neutral evolution.

S5 Impact of our simplified treatment of polymorphism

S5.1 Amount of polymorphism - theory versus simulation

Our theory for the overdispersed variation on the random GP map requires estimates of the amount of genetic diversity in an evolving population close to the monomorphic limit. In

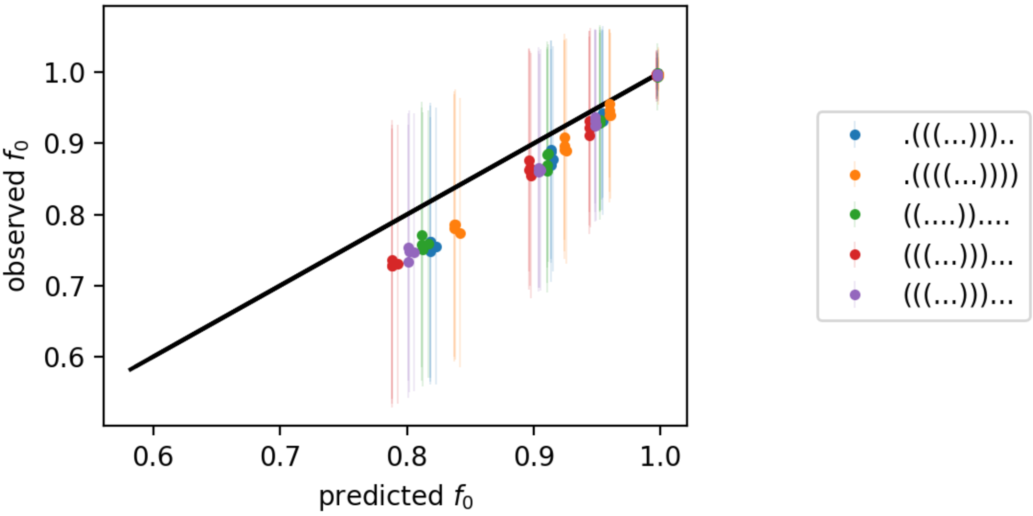

Figure S28: ***Comparing analytic estimates of the amount of polymorphism in a population to the simulation data:*** *The figure compares our analytic estimates of f_0 to simulation data. The simulations model neutral evolution (under stabilising selection) on the random maps corresponding to the NCs shown in Fig S5 above (structures shown in the legend). We use a range of population parameters, both monomorphic populations ($LuN \ll 1$) and populations with more genetic diversity ($LuN \approx 0.6$): population sizes $N = 10$, $N = 100$, $N = 1000$, $N = 2000$ and mutation rates $u = 0.0005/N$, $u = 0.01/N$, $u = 0.02/N$ and $u = 0.05/N$. The simulations are run for 10^6 generation, and f_0 is measured every 10^3 generations and averaged over; the error bars indicate the standard deviation in the measurements.*

Eq. S11, we derived the following approximation for the fraction of individuals f_0 that occupy the prevalent genotype:

$$f_0 \approx \sqrt{\frac{1}{1 + N(1 - uL(1-\rho))2uL\rho}} \tag{S24}$$

Fig S28 shows how this approximation compares to the measured value in simulations for a range of population parameters u and N. We find that the approximate values correlate with the simulation results and are of the right order of magnitude. This is "good enough" for our purposes, where we want to use a very simple treatment of the population to simplify the remaining calculations. However, it is also clear that even in this example, the approximation is not perfect, and it is expected to break down for highly polymorphic populations, which we are not considering in this paper. Furthermore, the population diversity in the simulations displays a high standard deviation and thus varies throughout the simulations, indicating that a single number cannot capture the full dynamics. Therefore, this treatment should not be applied to new situations without further tests.

S5.2 Impact of polymorphism terms in our calculations

Overall, the effect of the polymorphic corrections in our calculations is to reduce burstiness: we assume that mutations on the individuals in the 'polymorphic part' of the population (i.e. individuals which differ from the current prevalent genotype) can be described by a constant-rate model since the polymorphic part of the population is genotypically diverse. While such a simplifying assumption is required to make our further calculations feasible, it effectively underestimates the true burstiness of the process: the average-rate model would only be appropriate if the polymorphic part of the population was spread out evenly across the neutral space. In

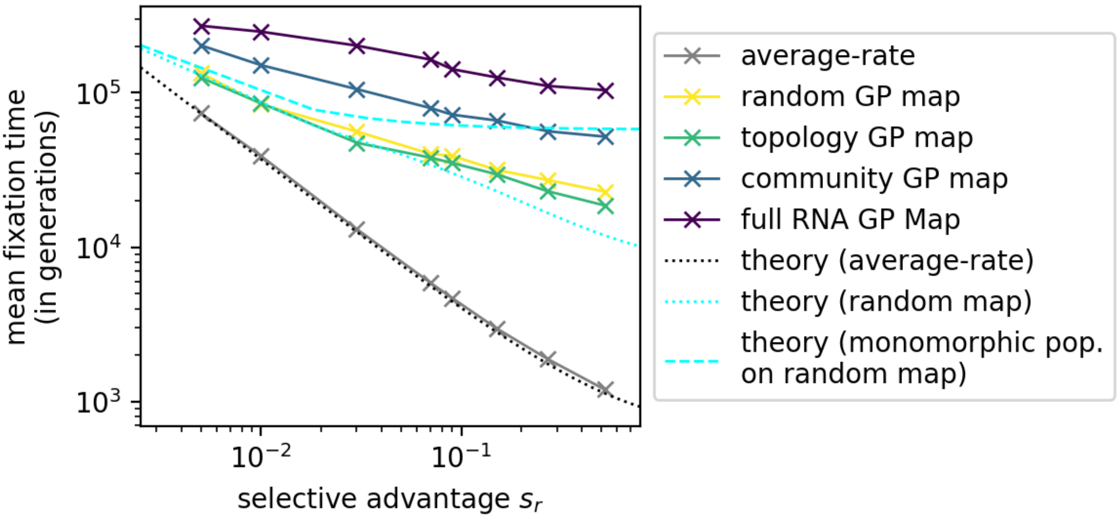

Figure S29: ***Impact of polymorphic terms on the predicted fixation time of highly adaptive phenotypes:*** *Since fixation times of highly adaptive (i.e. high-s_r) phenotypes are expected to be very sensitive to the approximations made when modelling the polymorphic part of the population, we plot two predictions together with the simulation data from Fig 6 in the main text: one with the polymorphic correction and one without this correction (i.e. for a purely monomorphic population with $f_0 = 1$). We find that the results are indeed sensitive to the treatment of the (small) polymorphic part of the population for high-s_r phenotypes. Since our calculations are likely to underestimate the burstiness in the 'polymorphic part' of the population, the truth should lie somewhere between the two extremes, and this expectation is in good agreement with the simulation data.*

reality, however, the polymorphic part of the population is concentrated on genotypes close to the prevalent genotype since we are considering cases close to the monomorphic limit, where only few mutations have typically been accumulated since the last common ancestor of any two individuals in the population. Thus, our calculations underestimate the burstiness in the (small) 'polymorphic part' of the population and predict that any accessible new phenotype p_i is produced at a low rate by the polymorphic part of the population, whereas in reality p_i should only be observed if the population's prevalent genotype is close to a portal genotype to p_i.
The effect of this simplification will be most prominent when the final result depends sensitively on the polymorphic part of the population. As discussed in section S1.3.4, this is the case for the fixation of highly adaptive mutants that are likely to fix after few introductions[1]. To quantify the impact of such inaccuracies, we repeated the analytic estimates of fixation times (Fig. 6 in the main text) without any polymorphic terms, i.e. with $f_0 = 1$ (see Fig. S29). We find that, as expected, our polymorphic correction lowers the estimated fixation time of highly adaptive (i.e. high-s_1) phenotypes compared to estimates without this correction. Since our approximations underestimate the burstiness in the 'polymorphic part' of the population, the truth should lie somewhere between the two extremes, and this expectation is in very good agreement with the simulation data.

[1]To be exact, this plays a role if $t_c \ll t_p$ with $t_c \approx (r_1(1 - f_0)P^{\text{fix}}_{p_1})^{-1}$. This can be applicable for highly polymorphic populations (with small f_0). It is also applicable if the single-mutant fixation probability $P^{\text{fix}}_{p_1}$ is high due to a high selective advantage s_1 of p_1

References

[1] E. Bornberg-Bauer and H. S. Chan. Modeling evolutionary landscapes: mutational stability, topology, and superfunnels in sequence space. *Proceedings of the National Academy of Sciences*, 96(19):10689–10694, 1999.

[2] M. C. Cowperthwaite, E. P. Economo, W. R. Harcombe, E. L. Miller, and L. A. Meyers. The ascent of the abundant: how mutational networks constrain evolution. *PLoS computational biology*, 4(7):e1000110, 2008.

[3] R. Dawkins. *The blind watchmaker : why the evidence of evolution reveals a universe without design. 30th anniversary edition.* Penguin Books, London, 2016. ISBN 9780141026169.

[4] K. Dingle, S. Schaper, and A. A. Louis. The structure of the genotype-phenotype map strongly constrains the evolution of non-coding RNA. *Interface Focus*, 5(6):20150053, 2015.

[5] K. Dingle, F. Ghaddar, P. Sulc, and A. A. Louis. Phenotype bias determines how RNA structures occupy the morphospace of all possible shapes. *Molecular Biology and Evolution*, 39(1):msab280, 2022.

[6] A. Etheridge. *Some Mathematical Models from Population Genetics: École d'Été de Probabilités de Saint-Flour XXXIX-2009.* Lecture Notes in Mathematics. Springer Berlin Heidelberg, Berlin, Heidelberg, 2011. ISBN 978-3-642-16632-7.

[7] S. F. Greenbury, S. Schaper, S. E. Ahnert, and A. A. Louis. Genetic correlations greatly increase mutational robustness and can both reduce and enhance evolvability. *PLOS Computational Biology*, 12(3):e1004773, 2016.

[8] A. A. Hagberg, D. A. Schult, and P. J. Swart. Exploring network structure, dynamics, and function using networkx. In G. Varoquaux, T. Vaught, and J. Millman, editors, *Proceedings of the 7th Python in Science Conference*, pages 11 – 15, Pasadena, CA USA, 2008.

[9] R. R. Hudson et al. Gene genealogies and the coalescent process. In D. Futuyma and J. Antonovics, editors, *Oxford Surveys in Evolutionary Biology*, number v. 7 in Oxford Surveys in Evolutionary Biology. Oxford University Press, 1991.

[10] E.-K. Kim and H.-H. Jo. Measuring burstiness for finite event sequences. *Physical Review E*, 94(3):032311, 2016.

[11] H. Li, R. Helling, C. Tang, and N. Wingreen. Emergence of preferred structures in a simple model of protein folding. *Science*, 273(5275):666–669, 1996.

[12] N. Martin and S. Ahnert. Thermodynamics and neutral sets in the RNA sequence-structure map. *Europhysics Letters*, 139(3):37001, 2022.

[13] N. S. Martin and S. E. Ahnert. The Boltzmann distributions of molecular structures predict likely changes through random mutations. *Biophysical Journal*, 122(22):4467–4475, 2023.

[14] N. S. Martin, C. Q. Camargo, and A. A. Louis. Bias in the arrival of variation can dominate over natural selection in Richard Dawkins's biomorphs. *PLOS Computational Biology*, 20 (3):e1011893, 2024.

[15] D. M. McCandlish. *Evolution on Arbitrary Fitness Landscapes When Mutation is Weak.* PhD thesis Duke University, 2012.

[16] D. M. McCandlish and A. Stoltzfus. Modeling evolution using the probability of fixation: history and implications. *The Quarterly Review of Biology*, 89(3):225–252, 2014.

[17] S. Schaper and A. A. Louis. The Arrival of the Frequent: How Bias in Genotype-Phenotype Maps Can Steer Populations to Local Optima. *PLoS ONE*, 9(2):e86635, 2014.

[18] M. Weiß and S. E. Ahnert. Neutral components show a hierarchical community structure in the genotype–phenotype map of RNA secondary structure. *Journal of the Royal Society Interface*, 17(171):20200608, 2020.

[19] M. Weiß and S. E. Ahnert. Using small samples to estimate neutral component size and robustness in the genotype–phenotype map of RNA secondary structure. *Journal of the Royal Society Interface*, 17(166):20190784, 2020.

[20] L. Y. Yampolsky and A. Stoltzfus. Bias in the introduction of variation as an orienting factor in evolution. *Evolution and Development*, 3(2):73–83, 2001.
